# Gut microbiota CLA and IL-35 induction in macrophages through Gαq/11-mediated STAT1/4 pathway: an animal-based study

**DOI:** 10.1080/19490976.2024.2437253

**Published:** 2024-12-05

**Authors:** Xiaomin Su, Yazheng Yang, Yunhuan Gao, Juanjuan Wang, Yang Hao, Yuan Zhang, Rongcun Yang

**Affiliations:** a Department of Immunology, Nankai University School of Medicine, Nankai University, Tianjin, China; b Translational Medicine Institute, Tianjin Union Medical Center of Nankai University, Tianjin, China; c State Key Laboratory of Medicinal Chemical Biology, Nankai University, Tianjin, China

**Keywords:** Conjugated linoleic acid, IL-35, macrophages, Reg4, gut microbiota

## Abstract

Gut microbiota/metabolites not only participate in the food and energy metabolism but also contribute to the host immune response and homeostasis. The alternation of gut microbiota/metabolites has been widely related to intestinal and extra-intestinal disorders such as intestinal bowel diseases (IBDs). Bactericidal substances from gut epithelial cells can regulate the composition of gut microbiota. However, the effects of regenerating protein 4 (REG4) (human)/(Reg4) (mice), a potentially bactericidal substance from gut epithelial cells, on the gut immune homeostasis maintain elusive. Here, we found that REG4/Reg4 is essential in maintaining gut immune homeostasis through REG4/Reg4 associated gut microbiota. Reg4 knockout (KO) mice were highly sensitive to DSS-mediated colitis, whereas human *REG4* intestine epithelial cell transgenic *(huREG4*^*IECtg*^) mice exhibited more resistance to DSS-mediated colitis. Mechanistically, sequencing of gut microbiota and liquid chromatography-mass spectrometry showed that REG4/Reg4 could affect the composition of gut microbiota. REG4/Reg4 associated gut microbiota such as *Lactobacillus* could metabolize linoleic acid (LA) into conjugated linoleic acid (CLA). Immunoprecipitation and immunoblot showed that CLA could effectively promote the expression of IL-35 in macrophages through Gα_q/11_ mediated activation STAT1/4. Thus, our results demonstrate that REG4/Reg4 plays a critical role in maintaining gut immune homeostasis through CLA-mediated IL-35^+^ macrophages.

## GRAPHICAL ABSTRACT

**Figure:**
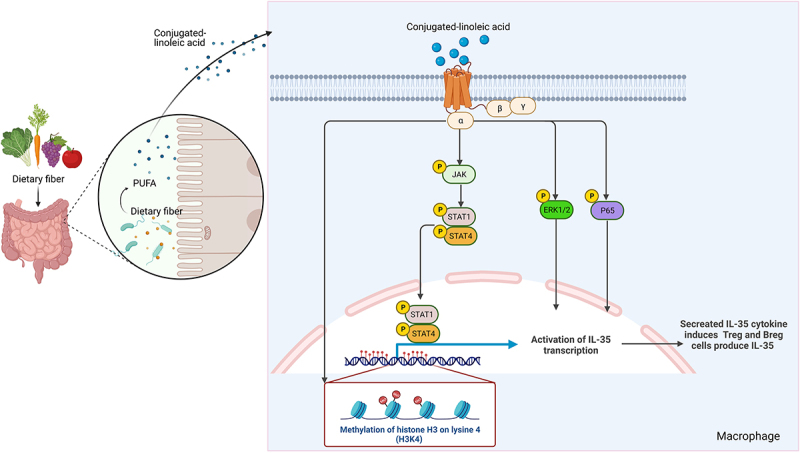

## Introduction

Inflammatory bowel disease (IBD) is a chronic relapsing-remitting disease with a remarkable increase in incidence worldwide.^1^ Importantly, IBDs, including ulcerative colitis (UC) and Crohn’s diseases (CDs), have a close relationship with developing colorectal cancer.^2^ Notably, the etiology and pathogenesis of IBD remain to be further clear.

Dysbiosis of gut microbiota is an important pathogenic factors for IBDs.^3^ Gut microbiota/metabolites not only participate in the food and energy metabolism but also contribute to the development of IBD,^4,5^ which depends on the immune cells in gut tissues. Immune cells such as immunosuppressive and inflammatory macrophages can express different receptors of gut microbiota/metabolites.^6–8^ Activation of these receptors affect the differentiation and function of immune cells, causing the reprogramming of local and systemic immune system for gut immune homeostasis^9,10^ through genetic and epigenetic regulation or metabolism of the immune cells.^11–13^ A substance of evidence has suggested an intricate relationship between the gut microbiota and the immune system.^10^ Importantly, most of altered gut microbiota mediated diseases are related to impaired immune responses.^10,14,15^

The gut immune responses are related to metabolites from gut microbiota. The conjugated linoleic acids (CLAs), a mixture of the positional and geometric isomers of linoleic acid (LA) such as *cis*-9 CLA and *trans*-10 CLA, are synthesized in the human and mouse colon.^16^ Although conjugated linoleic acids (CLAs) are found in food products,^17^ they mainly derive from the biohydrogenation of LA through bacteria that express LA isomerase.^18^ Various lactic acid bacteria, particularly *Lactobacillus* species, can produce high-purity *cis*-9 CLA and *trans*-10 CLA through enzymatic isomerization.^16,19^ These CLAs exhibit anti-carcinogenic, anti-atherogenic, anti-diabetic, and anti-obesity activity. They exert effects in part through membrane receptors such as G protein-coupled receptor (GPR)40 and GPR120,^20,21^ which were first described in the pancreas, intestinal cells,^22^ and the immune cells such as macrophages.^23^ Although there exists some knowledge on the impact of CLAs such as that CLAs affect the activity and mRNA expression of 5- and 15-lipoxygenases in human macrophages^24^ and regulate triacylglycerol and cholesterol concentrations in macrophages/foam cells by the modulation of CD36 expression,^25^ the effects of CLA on the macrophages are incompletely clear.

Macrophages mediated effects can be through cytokines such as interleukin-35 (IL-35), which is formed as a heterodimer composed of p35 (IL-12A) and Ebi3 (Epstein–Barr virus-induced gene 3).^26^ This cytokine has strong suppressive properties both *in vivo* and *in vitro*. This cytokine can be produced by immune cells such as macrophages, B and T cells.^27^ Notably, the generated IL-35 in the microenvironment can induce a potent regulatory T cell (Treg) and B cell (Breg) population,^28^ which play a critical role in maintaining immune homeostasis. IL-35-producing cells exert an essential role in the pathogenesis and development of some inflammatory diseases^27^ such as that *Ebi*3^−/−^ and *p35*^−/−^ mice are more sensitivity to colitis.^29,30^

Gut epithelial cells, which produce bactericidal substances such as regenerating gene (Reg) family members, play an important role in maintaining the homeostasis of gut microbiota.^31,32^ For instance, Reg3γ, a murine orthologue to human Reg3α, has bactericidal activity against Gram-positive pathogens.^33^ Importantly, enteric delivery of human Reg3α alters the intestinal microbiota and controls inflammation in mice with colitis.^34^ Reg4 expressed at gut mucosa can be detected in deep crypt of colon.^35^ It may bind to bacteria and induce damage to the bacterial cell wall^36^ and potentially affect the composition of gut microbiota.^31^ We previously found that Reg4 could induce changes in gut microbiota composition, causing increased *Lactobacillus*.^37^ With the the current limited knowledge on Reg4 regulation, we here explored how REG 4/Reg4 associated *Lactobacillus* maintained gut immune homeostasis in the colon tissues.

## Results

### REG4/Reg4 promotes resistance of mice to dss-mediated colitis

**Figure 1.**
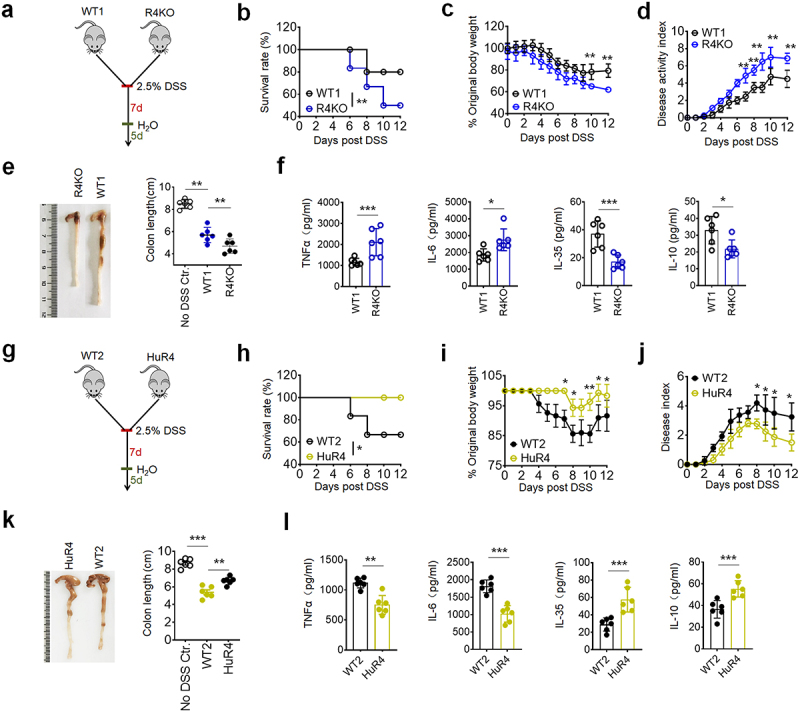
REG4/Reg4 resists to dss-mediated colitis. (a) The design for the dss-induced colon inflammation model for b-f. *Reg4* knockout (R4KO) and their littermate control mice (WT1) were exposed to 2.5 % DSS for 7 d, and then changed with normal water for 5 d in b, c and d; (b) survival rate of R4KO and WT1 mice; n = 18; (c) body weight of R4KO and WT1 mice; n = 6; (d) disease activity index (DAI) of R4KO and WT1 mice; n = 6; (e) colon length of R4KO and WT1 mice; n = 6; (f) ELISA of tnf-α, IL-6, IL-10 and IL-35 in the sera of R4KO and WT1 mice; (g) the design for the dss-induced colon inflammation model for h-l. *huREG4*^*IECtg*^ (HuR4) and littermate control mice (WT2) were exposed to 2.5 % DSS for 7 d, then and changed with normal water for 5 d in h, I and j or 2 d in k and l; (h) survival rate of HuR4 and WT2 mice; n = 18; (i) body weight of HuR4 and WT2 mice; n=6; (j) disease activity index (DAI) of HuR4 and WT2 mice; n = 6; (k) colon length of HuR4 and WT2 mice; n = 6; (l) ELISA of tnf-α, IL-6, IL-10 and IL-35 in the sera of HuR4 and WT2 mice. Wilcoxon’s test in b and h; one-way ANOVA test in c, d, e, I, j and k; Student’s *t*-test in f and l. *p < 0.05, **p < 0.05, ***p < 0.05. data is a representative of at least three experiments.

To investigate the roles of REG4/Reg4 in gut immune homeostasis, we employed a mouse model of dextran sodium sulfate (DSS)-mediated colitis (Figure 1a). Data showed that *Reg4* KO mice were more sensitive to DSS-mediated colitis, whereas *huREG4*^*IECtg*^ mice exhibited significantly stronger resistance as compared to the littermate controls (Figure 1). After exposure to 2.5% DSS in their drinking water for 7 d, these *Reg4* KO mice exhibited lower survival rate, more weight loss, higher disease index, and also more shortened colon as compared to the control mice (Figure 1a–e). There also had higher histology scores with stronger inflammation and more inflammatory cell infiltration in H&E staining in *Reg4* KO mice (Figure S1a). Inflammatory cytokines including TNFα and IL-6 were also higher in the colon lamina propria (CLP) of these *Reg4* KO mice. However, IL-35 and IL-10 immunosuppressive cytokines were lower in the CLP of the *Reg4* KO mice (Figure 1f). Inflammatory cells such as CD11b^+^Ly6C^+^, CD11b^+^ Ly6G^+^, CD4^+^IFNγ^+^Th1 cells and CD4^+^IL-17^+^ Th17 cells were much more in the CLP of *Reg4* KO mice (Figure S1b, Figure S2). In contrast to the *Reg4* KO mice, *huREG4*^*IECtg*^ mice showed markedly resistance to DSS-mediated colitis (Figure 1g–l, Figure S1c). There also had remarkably lower inflammatory cytokines with significantly reduced inflammatory cells and increased IL-35 and IL-10 immunosuppressive cytokines in *huREG4*^*IECtg*^ mice (Figure 1l, Figure S1d). Thus, REG4/Reg4 plays an important role in maintaining gut homeostasis.

### REG4/Reg4 mediated resistance to dss-induced colitis depends on IL-35

**Figure 2.**
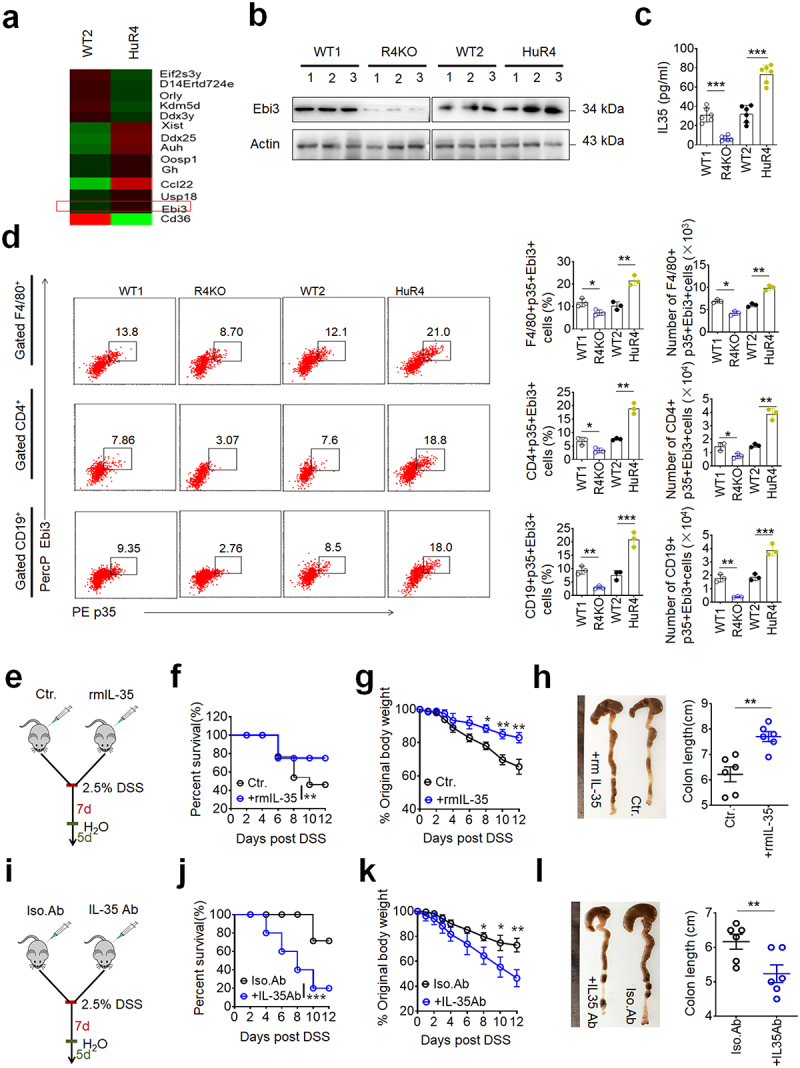
REG4/Reg4 mediated resistance to dss-mediated colitis is dependent on IL-35. (a) RNA-seq of colon lamina propria (CLP) tissues in *huREG4*^*IECtg*^ (HuR4) and their littermate control mice (WT2); (b) Western blotting of Ebi3 expression in colon lamina propria (CLP) tissues of R4KO, HuR4 and control mice. (n = 3); (c) ELISA of IL-35 in the sera of R4KO, HuR4 and control mice; (d) flow cytometry of F4/80^+^p35^+^Ebi3^+^, CD4^+^p35^+^Ebi3^+^ and CD19^+^p35^+^Ebi3^+^ cells in the CLP of R4KO, HuR4 and control mice. The proportion and absolute number F4/80^+^p35^+^Ebi3^+^, CD4^+^p35^+^Ebi3^+^ and CD19^+^p35^+^Ebi3^+^ cells in the colon were compared (n = 3); (e) the design for the effects of recommendation mouse IL-35 (rmIL-35) on R4KO mice on dss-mediated colitis for f-h; (f) survival rate of R4KO mice with (+rmIL-35) or without (ctr.) rmIL-35; n = 18; (g) body weight changes of R4KO mice with (+ rmIL-35) or without (ctr.) rmIL-35; n = 6; (h) colon length in of R4KO mice with (+rmIL-35) or without (ctr.) rmIL-35; n = 6; (i) the design for the effects of mouse IL-35 blocking antibody (IL-35Ab) on *huREG4*^*IECtg*^ mice on DSS mediated colitis for j-l; (j) survival rate of HuR4 mice with (+IL-35 ab) or without (isotypic control, Iso.Ab) IL-35 blocking antibody; n = 18; (k) body weight changes of HuR4 mice with (+IL-35 ab) or without (Iso.Ab) IL-35 blocking antibody; n = 6; (l) colon length of HuR4 mice with (+IL-35 ab) or without (Iso. Ab) IL-35 blocking antibody; n = 6. Wilcoxon’s test in f and j; one-way ANOVA test in g and k; Student’s *t*-test in c, d, h and l. *p < 0.05, **p < 0.05, ***p < 0.05. a representative of at least three experiments.

REG4/Reg4 can potentially be through multiple mechanisms to regulate sensitivity to DSS mediated colitis, such as gut cell development, gut immune cells and alteration of gut microbiota.^38^ Since we previously found REG4/Reg4 might cause increased IL-35^+^ immune cells through changed gut microbiota,^39^ we analyzed these IL-35^+^ cells in the CLP. Consistent with our previous data, there indeed were increased IL-35 cytokine and IL-35^+^ B immune cells in the CLP of *huREG4*^*IECtg*^ mice, and reduced IL-35 cytokine and IL-35^+^ B cells in *Reg4* KO mice (Figure 2a–d, Figure S3, S4). Further analyses showed that there were also reduced IL-35^+^ macrophages and IL-35^+^T cells in the CLP in *Reg4* KO mice; Whereas there were increased IL-35^+^ macrophages and T cells in *huREG4*^*IECtg*^ mice (Figure 2d). Increased Ebi and IL-35 subunits in the CLP of *huREG4*^*IECtg*^ mice also could be confirmed by RNA-Seq and immunoblot (Figure 2a,b).

IL-35, as an immune suppressive cytokine, plays a role in immune homeostasis. We further examined whether IL-35 determined the sensitivity of mice to DSS-mediated colitis in *Reg4* KO mice using recombinant mouse (rm) IL-35 and *huREG4*^*IECtg*^ mice using IL-35 neutralizing antibodies (Figure 2e, i). Results showed that rmIL-35 administration could indeed reduce the sensitivity of *Reg4* KO mice to DSS-mediated colitis, whereas IL-35 neutralizing antibodies could increase sensitivity in *huREG4*^*IECtg*^ mice (Figure 2e–I, Figure S5), consistent with the data that IL-35 reverses inflammatory bowel disease.^40^ Thus, our data clearly indicate that REG4/Reg4 mediated resistance to DSS-mediated colitis is dependent on IL-35.

### Gut microbiota derived CLA induces IL-35 production in macrophages

We next employed feces transplanted mouse model to investigate whether feces transplantation could affect the DSS-mediated colitis in *Reg4* KO or *huREG4*^*IECtg*^ mice (Figure S6). Interestingly, feces transplantation from wild-type (WT) to *Reg4* KO mice could reduce the sensitivity to DSS-mediated colitis; whereas the feces transplantation from WT to *huREG4*^*IECtg*^ mice could increase the sensitivity to DSS-mediated colitis (Figure S6a-h). Notably, immune cells such as IL-35^+^ T cells, IL-35^+^ B cells, and IL-35^+^ macrophages also increased in the WT feces transplanted *Reg4* KO mice; Whereas there were reduced IL-35 cytokine and decreased IL-35^+^T and B cells, and IL-35^+^ macrophages in the WT feces transplanted *huREG4*^*IECtg*^ mice (Figure S6g, h). To further confirm this, feces from *Reg4* KO, *huREG4*^*IECtg*^ mice and control mice were also transplanted into germ-free (GF) mice, and the results showed that IL-35^+^ T and B cells, and IL-35^+^macrophages were indeed related to REG4/Reg4 gut microbiota (Figure S6I). Taken together, REG4/Reg4 mediated IL-35 is dependent on the gut microbiota.

**Figure 3.**
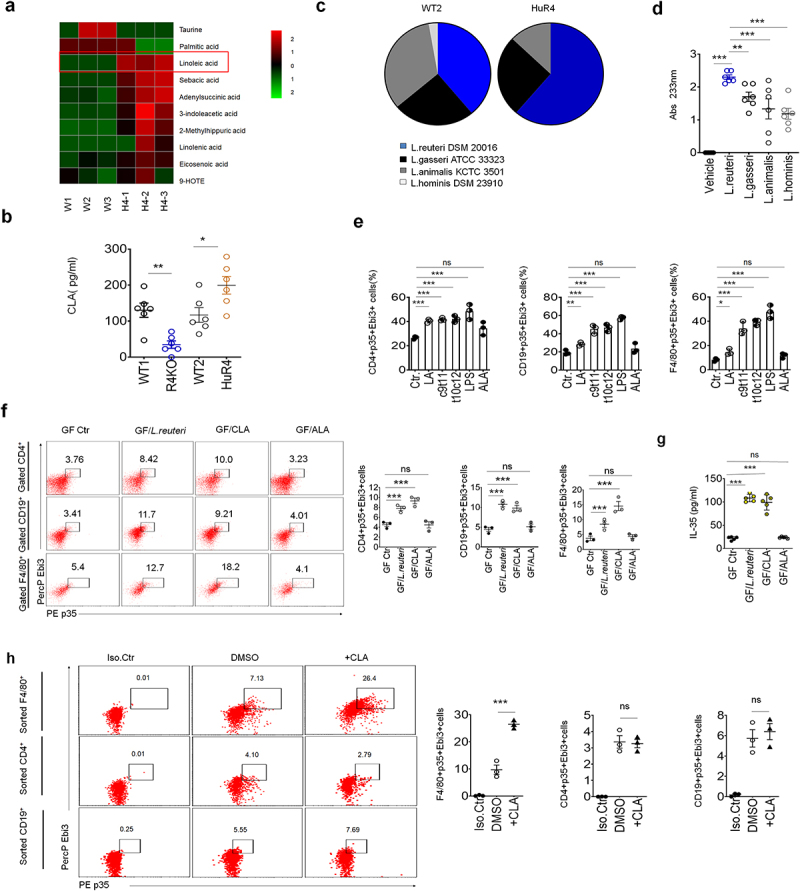
Gut microbiota derived CLA induces IL-35 production in macrophages (a) LC-MS of the sera from *huREG4*^*IECtg*^ (H4–1, H4–2 and H4–3) and their littermate control mice (W1, W2 and W3); (b) analyses of conjugated linoleic acid (CLA) in the sera of R4KO, HuR4 and their control mice; (c) proportion of different *Lactobacilli* in the colon contents of WT2 and HuR4 mice; (d) the levels of conjugated linoleic acid (*cis*-9 and *trans*-11 isomes) in the supernatants of different *Lactobacillus* species. *L. reuteri*, *Lactobacillus Reuteri* isolated from HuR4 mice; *L.Animalis*, *Lactobacillus animalis* (BNCC, China); *L. gasserri*, *Lactobacillus gasserri* (BNCC, China); *L.Hominis*, *Lactobacillus hominis* (BNCC, China); (e) flow cytometry of CD4^+^p35^+^Ebi3^+^, CD19^+^ p35^+^Ebi3^+^ and F4/80^+^ p35^+^Ebi3^+^ cells after exposure to vehicle, LA, CLA1 (c9t11, *cis*-9 CLA), CLA2 (t10c12, *trans*-10 CLA) and LPS, ALA (adenylsuccinic acid); (f) flow cytometry of CD19^+^p35^+^Ebi3^+^, CD4^+^p35^+^Ebi3^+^ and F4/80^+^p35^+^Ebi3^+^ cells in the colon lamina propria (CLP) tissues from GF mice with *L. reuteri* (GF/*L.Reuteri*), or CLA (GF/CLA), or ALA(GF/ALA) gavage or without (GF ctr) treatment; (g) ELISA of IL-35 in the colon tissues of GF mice with *L.Reuteri* (GF/*L.Reuteri*), CLA (GF/CLA), ALA(GF/ALA) gavage or without (GF ctr) treatment in vivo; (h) flow cytometry of p35^+^Ebi3^+^ cells in sorted CD4^+^ T cells, CD19^+^ B cells or F4/80^+^ macrophages with CLA(+CLA, *cis*-9 LA) or without (DMSO). Iso.Ctr, isotype IgG control. One-way ANOVA test; *p < 0.05, **p < 0.05, ***p < 0.05. a representative of at least three experiments.

Gut microbiota/metabolites can cause the reprogramming of local and systemic immune system to maintain gut immune homeostasis.^4,5^ We next analyzed the composition of gut microbiota metabolites again. Beyond the previously reported 3-indoleacetic acid (IAA) by us,^39^ there also existed other several increased metabolites such as LA in the blood of *huREG4*^*IECtg*^ mice by non-targeted metabolomic lipid chromatography-tandem mass spectrometry (LS-MS) (Figure 3a). Further studies showed that conjugated LA (CLA) were markedly higher in the peripheral blood of these *huREG4tg* mice; Whereas there was lower in Reg4 KO mice (Figure 3b). Meanwhile, we also determined whether increased *Lactobacillus* in *huREG4*^*IECtg*^ mice^39^ could metabolize LA to CLA (Figure 3c). In vitro culture, isolated dominant strain *L. Rutteri*^39^ could induce LA to produce more conjugated linoleic acid (mainly *cis*-9 and *trans*-11 isomer) (Figure 3d), consistent with other reports.^41,42^ CLAs, such as *cis*-9 and *trans*-10 CLA isomers,^43^ are related to multiple diseases, such as cancer, inflammation, and autoimmune diseases.^44^ To observe the effects of gut microbiota metabolite CLA on colonic inflammation, we set up coculture of mouse spleen cells to detect IL-35 expression in the immune cells in the presence of CLA. The results showed that CLAs *cis*-9 and *trans*-10 isomers could induce IL-35^+^ cells (Figure 3e, Figure S7). Notably, LA could also have a role in inducing the expression of IL-35 in different immune cells; whereas adenylsuccinic acid (ALA) did not have effects (Figure 3e). LPS, as a positive control, also induced the production of IL-35^45^ (Figure 3e). Notably, *in vivo* experiments, there were increased IL-35^+^ cells in colon tissues (Figure 3f) and higher IL-35 concentration in the serum of germ-free (GF) mice by CLA *cis*-9 isomer gavage (Figure 3g), suggesting that CLA can indeed induce the production of IL-35. Interestingly, *in vitro* culture for 16 h, CLA *cis*-9 isomer could only induce IL-35 in the macrophages but not T and B cells, suggesting that *cis*-9 CLA could directly induce the IL-35 in macrophages but not in T and B cells (Figure 3h). Thus, CLA from gut microbiota *Lactobacillus* of *huREG4*^*IECtg*^ mice could induce IL-35 cytokine in the macrophages through CLA.

**Figure 4.**
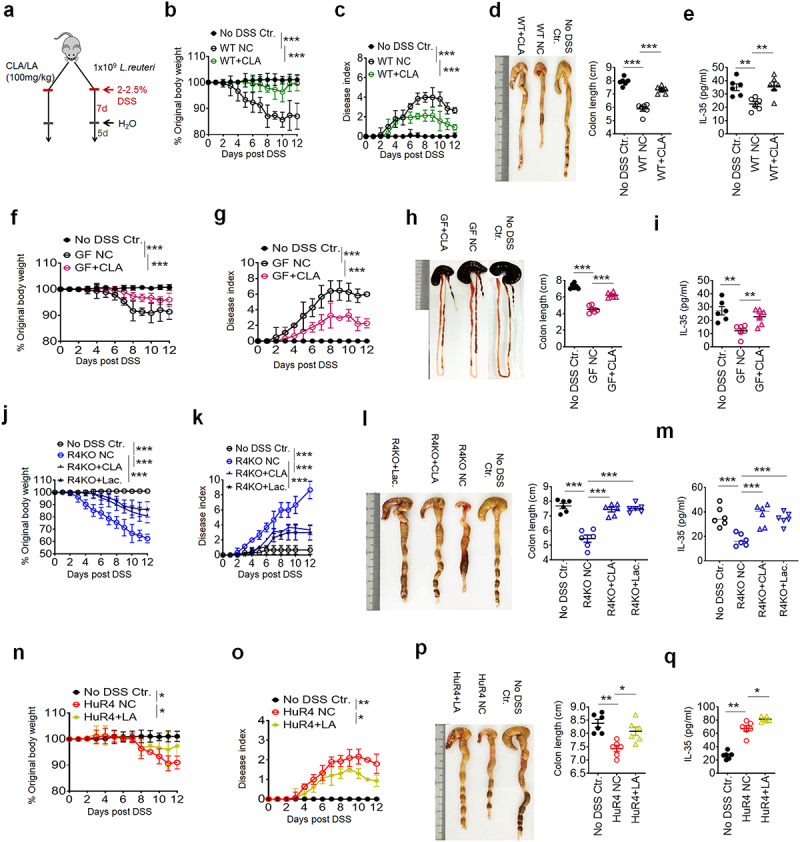
CLAs reduce sensitivity to DSS mediated colitis. (a) A schematic illustration showing the design for CLA/*L.Reuteri* gavage studies. (b) Body weight changes; n = 6; (c) disease index; n = 6; (d) colon length; n = 6 (e) ELISA of IL-35 in sera; wild type (WT) mice were treated with 100mg/kg CLA (WT+CLA) gavage then given 2.5% DSS for 7 d, and normal drinking water for another 5 d; (f) body weight changes; n = 6; (g) disease index; n = 6; (h) colon length; n = 6; (i) ELISA of IL-35 in sera; germ-free (GF) mice were treated with 100mg/kg CLA (GF+CLA) gavage, and then with 2% DSS; (j) body weight changes; n = 6; (k) disease index; n=6; (l) colon length; n = 6; (m) ELISA of IL-35 in sera; *Reg4* knockout (R4KO) mice and littermate control mice were treated with 100mg/kg CLA (R4KO+CLA) or with 1 × 10^9^ *L.Reuteri* (R4KO+Lac) gavage, and then with 2.5% DSS; (n) body weight changes; n = 6; (o) disease index; n = 6; (p) colon length; n = 6; (q) ELISA of IL-35 in sera; *huREG4*^*IECtg*^ (HuR4). Mice were treated with 100mg/kg LA (R4KO+LA) gavage, and then given with 2.5% DSS. NC, PBS control group under DSS colitis model. No DSS ctr., no DSS and no other treatment group. One-way ANOVA test; *p < 0.05, **p < 0.05, ***p < 0.05. a representative of at least two experiments.

Next, we examined the effects of CLA on the DSS-mediated colitis, and mice were first treated by *cis*-9 CLA gavage, and then fed using 2.5% DSS for 7 d (Figure 4a). Data showed that *cis*-9 CLA could significantly reduce the DSS-mediated inflammation (Figure 4b–d) and increased production of IL-35 in the CLP (Figure 4e). Gavage of *cis*-9 CLA into GF mice could also markedly reduce sensitivity to DSS-mediated colitis (Figure 4f–h) and promoted the production of IL-35 in the CLP (Figure 4i). We also found that *cis*-9 CLA gavage could promote the resistance of *Reg4* KO mice to the DSS-mediated colitis (Figure 4 j–l). Gavage of *L. reuteri*, which can produce CLA, also reduced sensitivity to DSS mediated sensitivity in *Reg4* KO mice (Figure 4 j–l, Figure S8). Increased IL-35 in the CLP could also be observed in the *Reg4* KO mice treated by CLA or *Lactobacillus* gavage (Figure 4m). The administration of LA in *huREG4*^*IECtg*^ mice, which had enough *Lactobacillus* to convert LA to CLA, also reduced sensitivity to DSS-mediated colitis (Figure 4n–p). Furthermore, IL-35 levels in the CLA of *huREG4*^*IECtg*^ mice administering LA are also markedly upregulated (Figure 4q). Thus, gut microbiota *Lactobacillus* metabolites CLA can resist DSS-mediated colitis.

### Cla-mediated IL-35 production is through activating STAT1/4 in macrophages

**Figure 5.**
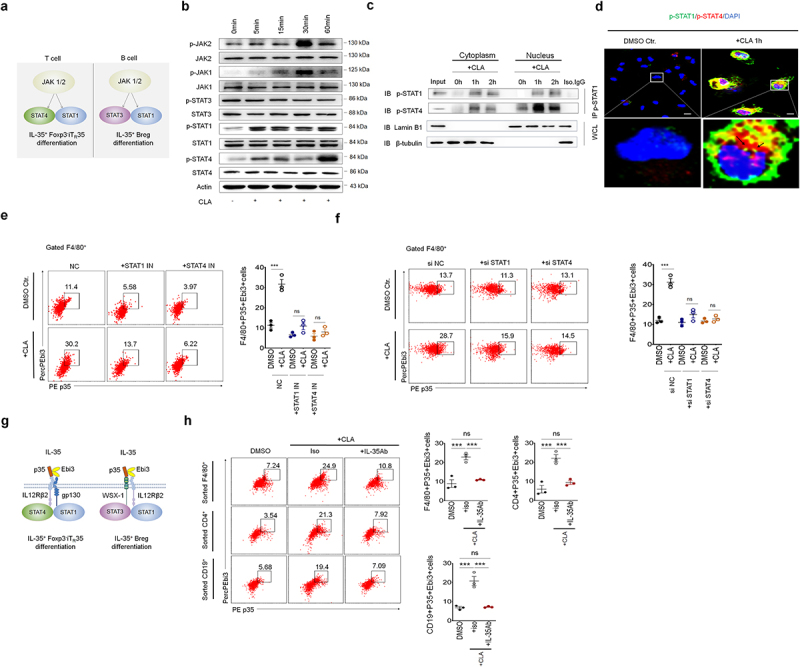
CLA mediated IL-35 production is through activating STAT1/4 in macrophages. (a) JAK-stat signal pathway is involved in inducing IL-35^+^ T and B cells; (b) immunoblotting of phosphor (p)-JAK2/JAK1/STAT1/STAT3/STAT4 and total JAK2/JAK1/STAT1/STAT3/STAT4) in the bone marrow derived macrophages (BMDMs) upon exposure to *cis*-9 CLA at the indicated time. Actin, loading control; (c) immunoblotting of p-STAT1 and p-STAT4 in the BMDMs after immunoprecipitated with anti-p-STAT1. WCL, whole cell lysis. LaminB1 and β-tubulin, loading control in nucleus and cytoplasm respectively; (d) immunostaining of p-STAT1(green) and p-STAT4(red) in the BMDMs with (+ cis-9 CLA 1h) or without (DMSO ctr.) CLA stimulation. Macrophages were observed by confocal microscopy with an oil immersion objective of 40 × and a digital zoom of 3. Nucleus was stained with DAPI (blue). Arrows indicated binding sites; scale bar=10 μM; (e) flow cytometry of F4/80^+^p35^+^Ebi3^+^cells in the BMDMs. BMDMs were pre-exposed to STAT1 inhibitor (+STAT1IN) or STAT4 inhibitor (+STAT4IN) for 6 h, and then cells were stimulated with (+ *cis*-9 CLA) or without (DMSO. Ctr) CLA for 24hrs; (f) flow cytometry of F4/80^+^p35^+^Ebi3^+^cells in BMDMs after transfected with STAT1 siRNA (+siSTAT1) or STAT4 siRNA (+siSTAT4) for 24hrs, and then stimulated with (+*cis*-9 CLA) or without (DMSO. Ctr) CLA for 24hrs; (g) classical signal molecules which is involved in IL-35 mediated IL-35^+^ Foxp3-iT_R_35 differentiation and IL-35^+^ Breg differentiation; (h) flow cytometry of p35^+^Ebi3^+^cells in the co-culture of CD4^+^ and CD19^+^ cells with F4/80^+^cells upon exposure to CLA (+CLA) with IL-35 antibody (+IL-35Ab) or isotypic antibody (+iso) for 24hrs. One-way ANOVA test; *p < 0.05, **p < 0.05, ***p < 0.05. a representative of at least two experiments.

The interaction of STAT4 with STAT1 may cause IL-35^+^ B cells^46,47^ (Figure 5a). Thus, we first examined the activation of isoSTAT in macrophages upon exposure to *cis*-9 CLA. Immunoblotting showed that the *cis*-9 CLA could effectively activate STAT1/4 in the macrophages (Figure 5b, Figure S9). Immunoprecipitation and immunostaining exhibited the binding of STAT1 and STAT4 (Figure 5c, d, Figure S9). Meanwhile, JAK1 and JAK2 were also activated after exposure to *cis*-9 CLA (Figure 5b). In addition, we also found that *cis*-9CLA could activate ERK1/2, p38 MAPK and NF-κBp-65 in the macrophages (Figure S10), consistent with other reports.^23^ Interestingly, silencing STAT1/4 or STAT1/4 inhibitors could affect the expression of IL-35 (Figure 5e,f), and *cis*-9 CLA mediated activation of STAT1/4 in the macrophages (Figure S11a, b). Taken together, all of these suggest that STAT1/4 was involved in the IL-35 production in the macrophages.

Previous studies found that IL-35 in the environment could not only induce IL-35 expression in the self-cells but also other immune cells^48,49^ through interaction of isoSTAT (Figure 5g). Since our studies demonstrated that *cis*-9 CLA could cause IL-35 production in the macrophages but not in B and T cells (Figure 3h), we next investigated the role of IL- 35 derived from macrophages in the T and B cells. Indeed, IL-35 antibody could markedly inhibit the IL-35 production in T and B cells in the coculture of macrophages with T and B cells after exposed to *cis*-9 CLA (Figure 5h). Thus, the macrophages derived IL-35 also cause IL-35 expression in T and B cells.

### CLA mediated activation STAT1/4 is through GPR isoform Gα_q/11_ chain in macrophages

**Figure 6.**
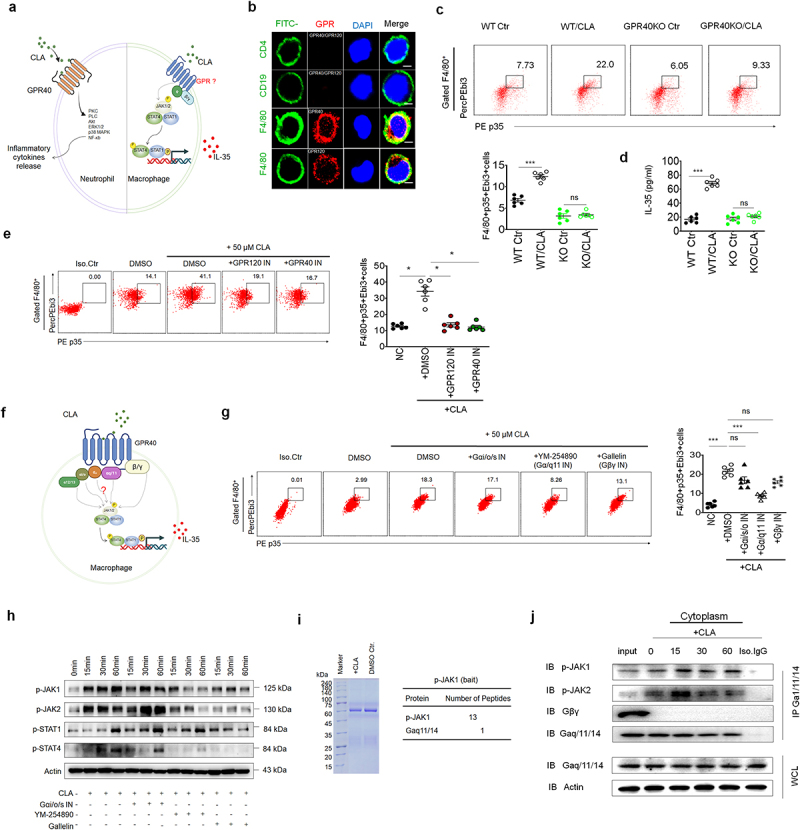
CLA mediated STAT1/4 activation is through GPR Gα_q/11_. (a) Conjugated linoleic acid (CLA) stimulates signaling pathways via the GPR40 receptor in neutrophil but it is unclear in macrophages; (b) immunostaining of GPR40/GPR120 (red) in CD4^+^ T cells, CD19^+^B cells and F4/80^+^ macrophages (green). Macrophages were observed by confocal microscopy with an oil immersion objective of 40 × and a digital zoom of 3. DAPI (blue) for nuclear staining; scale bar =10 μM; (c) flow cytometry of F4/80^+^p35^+^Ebi3^+^ cells of colon lamina propria (CLP) tissues from *GPR40* -/- mice (GPR40KO) or control mice(wt) with CLA (CLA) or without (Ctr) CLA gavage; (d) ELISA of IL-35 in sera from *GPR40* -/- mice(ko) or control mice(wt) with CLA (*cis*-9 CLA) or without (Ctr) CLA gavage; (e) flow cytometry of F4/80^+^p35^+^Ebi3^+^cells in BMDMs after exposure to CLA (+50μM CLA, DMSO) with GPR40 inhibitor (50μM CLA+GPR40IN) or GPR120 inhibitor (50μM CLA+GPR120IN) for 24hrs. Iso. Ctr, isotype control. DMSO, unstimulated control; (f) G-protein subunits including α12/13、αi/o、αs、αq/11 and βγ, which were potentially involved in the production of IL-35 by CLA in macrophages. (g) Flow cytometry of F4/80^+^p35^+^Ebi3^+^cells in the BMDMs after exposed to CLA (50μM CLA, DMSO) with Gαi/o/s inhibitor (50μM CLA+Gαi/o/sIN) or Gα/q11 inhibitor YM-254890 (50μM CLA + Ga/q11IN) or Gβγ inhibitor gallelin (50μM CLA + GβγIN) for 24hrs. Iso. Ctr, isotype control. DMSO, unstimulated control; (h) immunoblotting of p-JAK1/JAK2/STAT1/STAT4 in BMDMs exposed to CLA with Gαi/o/s inhibitor (Gαi/o/sIN +), Gα/q11 inhibitor (YM-254890+) or Gβγ inhibitor (galletin+) at indicated time. (-), no stimulated control. Actin, a loading control; (i) interaction of Gα/q11(also known as Gα/q11/14) with phospho-JAK1 after CLA stimulation. BMDMs were exposed to CLA (+CLA) or DMSO control (Dmso.Ctr), and then immunoprecipitated with anti-phospho-JAK1; (j) immunoblotting of p-JAK1/JAK2, Gβγ and Ga/q11/14 in the BMDM lyses immunoprecipitated with anti-Ga/q11/14. WCL, whole cell lysis; IP, immunoprecipitation; IB, immunoblotting; Iso. IgG, isotype IgG control. One-way ANOVA test; *p < 0.05, **p < 0.05, ***p < 0.05. a representative of at least two experiments.

GPR40 and GPR120 can activate the heterotrimeric G protein^23,50^ (Figure 6a). Thus, we first examined the expression of CLA receptor in the macrophages. Results showed that macrophages but not B and T cells could express GPR40 and GPR120, consistent with other reporters^23^ (Figure 6b). After *cis*-9 CLA gavage, GPR40 KO mice did not cause increased IL-35^+^ macrophages (F4/80^+^p35^+^Ebi^+^ cells) (Figure 6c) and also IL-35 in the CLP (Figure 6d). However, GPR120 also played a role in CLA mediated production of IL-35 in the macrophages. GPR120 inhibitor reduced the production of IL-35^+^ macrophages (Figure 6e). These results suggest that the CLA mediated IL-35 is through GPR 40 and GPR120.

GPR40 and/or GPR120 could activate the heterotrimeric G protein composed of α, β and γ chain^23,50^ (Figure 6f). There are multiple isoforms such as Gα_q/11_, Gα12/13, G_s_α, and Gαi/o in α chain, which exert different functions in mediating intracellular signaling^51^ (Figure 6f). Using α subunits inhibitor YM-254890, we found that this inhibitor not only affected the production of IL-35 but also STAT1/4 and JAK1/JAK2 phosphorylation, whereas β- and γ-chain inhibitor gallelin^52^ did not produce similar effects (Figure 6g,h), indicating that Gα_q/11_ chain of heterotrimeric G proteins plays a critical role in inducing IL-35 expression and STAT1/STAT4 activation in the macrophages. Similar effects did not happen in α-chain Gαi/o inhibitor rimonabant treated cells, suggesting that α-chain Gαi/o were not involved in the IL-35 expression and activation of STAT1 and STAT4 (Figure 6g,h). Since limited Gα12/13 and Gαs inhibitors, we here only tested G α_q/11_ (YM-254890) and Gαi/o (Rimonabant) inhibitors.^53^ Thus, G protein Gα_q/11_ is involved in CLA mediated IL-35 expression and activation of STAT1/4.

Next, we analyzed factors which could directly cause the activation of STAT1/4 activations. JAK/STAT signaling is a typical signal pathway.^54^ JAKs (JAK1, JAK2, JAK3, and TYK2) can mediate receptor tyrosine phosphorylation and recruit ≥1 STAT proteins (STAT1, STAT2, STAT3, STAT4, STAT5a, STAT5b, and STAT6).^55^ Results demonstrated that CLA could activate JAK2 and JAK1 (Figure 6h). JAK1 and JAK2 activation could also be affected by Gα_q/11_ subunit inhibitor YM-254890 (Figure 6h). Importantly, immunoprecipitation demonstrated the binding of JAK1/2 and Gα_q/11_ (Figure 6i,j). Thus, REG4 associated gut microbiota CLA can induce IL-35 expression in macrophages through receptor Gα_q/11_ mediated activation of JAK1 and JAK2. Finally, we also examined the effect of CLA on the enrichment of H3K4me3 on the promoter region of IL-35 subunit Ebi3. Results showed CLA could promote the enrichment of H3K4me3 on the promoter region of IL-35 Ebi3 (Figure S12). Thus, our data demonstrate that CLA not only induces activation of JAK1/2 and STAT1/4 but also affects the enrichment of H3K4me3.

Taken together, CLA can promote the expression of IL-35 in macrophages through the receptor isoform Gα_q/11_ mediated activation JAK1/2 and STAT1/4 and the enrichment of H3K4me3 in the macrophages.

### CLA-mediated IL-35 in human macrophages is through a similar mechanism with mice

**Figure 7.**
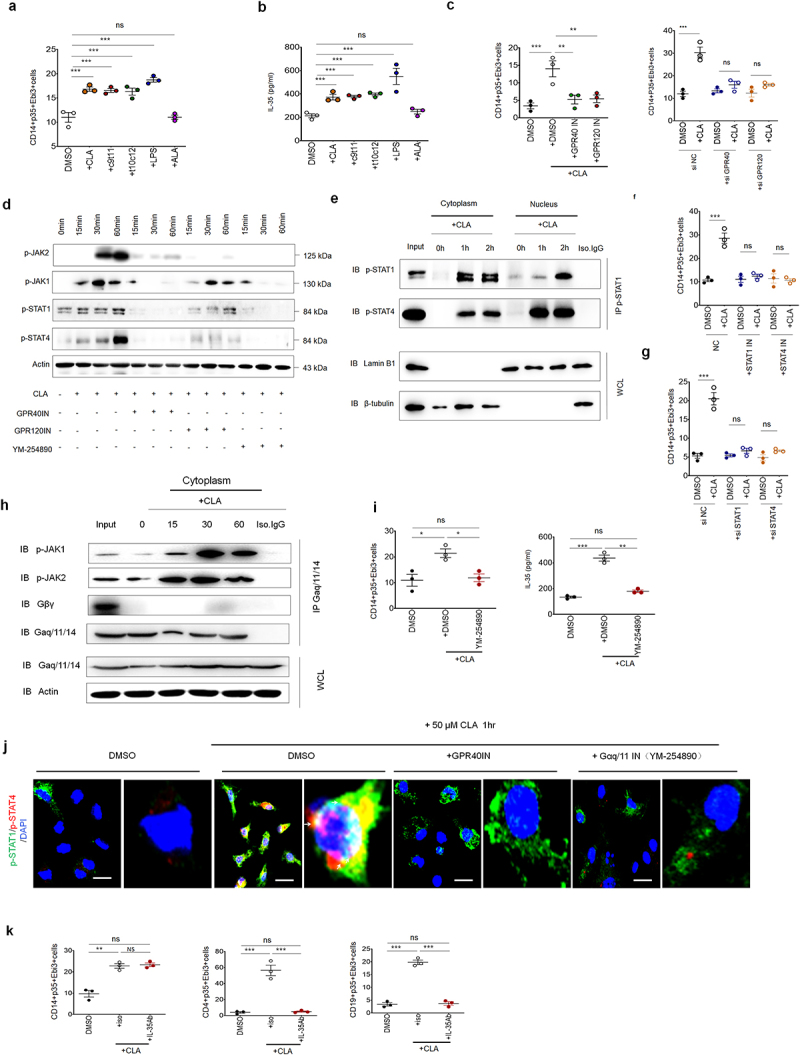
CLA induces IL-35 expression in human monocytes/macrophages. (a)Flow cytometry of CD14^+^p35^+^Ebi3^+^cells in the human peripheral blood monocyte cells derived macrophages (BMDMs) upon exposure to CLA (+CLA), c9t11 (cis-9 CLA), t10c12 (*trans*-10 CLA), ALA(+ALA) or LPS for 24hrs. DMSO, unstimulated control; (b) ELISA of IL-35 in the supernatants of BMDMs upon exposure to CLA (+CLA), c9t11 (*cis*-9 CLA), t10c12 (*trans*-10 CLA), ALA(+ALA) or LPS for 24hrs. DMSO, unstimulated control; (c) flow cytometry of CD14^+^p35^+^Ebi3^+^ in the BMDMs upon exposure to CLA(+CLA) with GPR40 inhibitor (CLA+GPR40IN) or GPR120 inhibitor (CLA+GPR120IN) for 24 h or GPR40 (si GPR40) or GPR120 (si GPR120) siRNAs or control (si NC) for 3 d. DMSO, unstimulated control; (d) immunoblotting of p-JAK2/JAK1/STAT1/STAT4 in the BMDMs upon exposure to CLA with GPR40 inhibitor (GPR40IN), GPR120 inhibitor (GPR120IN) or Gα/q11 inhibitor (YM-254890) at the indicated time. Actin as the loading control; (e) immunoblotting of p-STAT1 and p-STAT4 in the BMDM after immunoprecipitation with anti-p-STAT1. WCL: whole cell lysis, Lamin B1 and β-tubulin, loading control in nucleus and cytoplasm respectively; (f) flow cytometry of CD14^+^p35^+^Ebi3^+^cells in the BMDMs. Cells were pre-exposure to STAT1 inhibitor (+STAT1IN) or STAT4 inhibitor (+STAT4IN) for 6 h and then stimulated with (+CLA) or without (DMSO. Ctr) CLA for 24hrs; (g) flow cytometry of CD14^+^p35^+^Ebi3^+^cells in the BMDMs. Cells were transfected with STAT1 siRNA (+siSTAT1) or STAT4 siRNA (+siSTAT4) for 24hrs, and then stimulated with (+CLA) or without (DMSO. Ctr) CLA for 24hrs; (h) immunoblotting of phospho-JAK1/JAK2, Gβγ and Gα_q/11_ in the BMDM after immunoprecipitation with anti- Gα_q/11_. WCL, whole cell lysis; IP, immunoprecipitation; IB, immunoblotting; Iso.IgG, isotype IgG control; (i) flow cytometry of CD14^+^p35^+^Ebi3^+^cells in BMDMs and ELISA of IL-35 in the supernatant of BMDMs upon exposure to CLA (+CLA) with or without Gα_q/11_ (DMSO) inhibitor for 24hrs; (j) immunostaining of p-STAT1(green) and p-STAT4(red) in the BMDMs with (50μM CLA 1h) or without (DMSO) CLA stimulation. Nuclear was stained with DAPI (blue). Arrows indicated binding sites. (k) Flow cytometry of CD14^+^p35^+^Ebi3^+^cells, CD4^+^p35^+^Ebi3^+^cells or CD19^+^p35^+^Ebi3^+^cells in the coculture of the BMDMs with CD4^+^ and CD19^+^cells upon exposure to CLA (+CLA) with (+IL-35Ab) or without (isotypic antibody, iso) IL-35 antibody for 24hrs; one-way ANOVA test; *p < 0.05, **p < 0.05, ***p < 0.05. a representative of at least two experiments.

Since GPR40 and GPR120 could be detected in human macrophages,^56,57^ we next determined whether CLA could also induce IL-35 expression in human macrophages. Human macrophages were generated from peripheral blood monocytes and then exposed to CLAs. As expected, data showed that CLA, *cis*-9 and *trans*-10 CLA isomers could induce IL-35^+^ expression in the macrophages (Figure 7a,b). Notably, GPR40 or GPR120 inhibitors, and siRNAs targeting GPR40 or GPR120 could also limit the production of IL-35 upon exposure to *cis*-9 CLA (Figure 7c), indicating the involvement of both receptors. Upon exposure to *cis*-9 CLA, both STAT1 and STAT4 could be activated by *cis*-9 CLA (Figure 7d). GPR40 or GPR120 inhibitor also affected activation of STAT1 or STAT4 in human macrophages (Figure 7d). Further studies showed that CLA induced the interaction of STAT1 and STAT4 in human macrophages (Figure 7e). Silencing STAT1/4 or STAT1/4 inhibitor could also affect the production of IL-35^+^ macrophages and IL-35 production (Figure 7f,g), and CLA mediated activation of STAT1/4 in human macrophages (Figure S13). Furthermore, immunoprecipitation indicated the binding of G_q/11_α with JAK1 and JAK2 (Figure 7h). Gα_q/11_ inhibitor also affected the production of IL-35^+^ macrophages and the expression of IL-35 cytokine (Figure 7i). Immunostaining exhibited not only the interaction of STAT1 and STAT4, but also the effects of GPR40 and Gα_q/11_ inhibitor on the binding of STAT1/4 (Figure 7j). In addition, CLA could increase the enrichment of H3K4me3 on the promoter region of Ebi3 in M-CSF induced human macrophages (Figure S14). Finally, we also demonstrated that the expression of IL-35 in human T and B cells also depended on the macrophage-derived IL-35 in the coculture of macrophages with T and B cells (Figure 7k). Similar to mice, all of these results suggest that CLA can also induce IL-35 in human macrophages through Gα_q/11_ mediated activation of JAK1/2 and STAT1/4, as well as enrichment of H3K4me3 on the promoter region of Ebi3.

## Discussion

We found that the gut epithelial bactericidal substance REG4/Reg4 can promote the homeostasis of the gut immune system through altered gut microbiota. Indeed, dysregulated production of bactericidal substances is associated with IBD.^58,59^ The bactericidal substances from gut epithelial cells can suppress intestinal inflammation.^34^ The deficiencies in bactericidal substances may promote development of IBD. Our previous studies showed that REG4/Reg4 could prevent the overgrowth of *E. coli* in the mouse colon,^37^ which could cause colonic inflammation. Recent studies also found that the loss of colonic anti-microbial peptides could promote dysbiotic Gram-negative inflammaging-associated bacteria in aging mice.^60^ Interestingly, the expression of both *REG4* mRNA and protein was significantly upregulated in the colonic mucosa of UC patients in remission.^61^ Thus, the levels of REG4/Reg4 expression in the gut are important in maintaining gut immune homeostasis. Notably, several studies found the four genes (*REG Iα*, *REG Iβ*, *HIP/PAP*, and *REG IV*) were overexpressed in IBD samples.^62–64^

The macrophages exhibited a reduced inflammatory profile in response to both *cis*-9, *trans*-11-CLA and *trans*-10, *cis*-12-CLA.^65^ CLA was also shown to promote an M2 phenotype in cultured macrophages.^65^ 10,12 CLA specifically has been recently shown to promote the enrichment of adipose tissue with M2 macrophage markers arginase-1 (ARG-1) and early growth response protein-2 (EGR-2) in mice.^65^ CLA could also blunt the cluster of differentiation 18 (CD18) surface expression and CXC chemokine receptor 4 (CXCR4) expression on human peripheral blood mononuclear cells, resulting in suppressed monocyte adhesion in a mouse model of atherosclerosis.^66^ Our data clearly indicate that REG4/Reg4 mediated resistance to DSS-mediated colitis is dependent on IL-35, which is produced in the macrophages through CLA from gut microbiota. Collectively, CLA isomers are through multiple mechanisms to exert anti-inflammatory effects on monocytes and macrophages.

CLAs mediated IL-35 expression is through GPRs such as GPR40 and GPR120 mediated activation of STAT1/4 in the macrophages. CLA can activate wide signaling pathways to mediate immune cell functions.^23^ Previous reports showed that combination of different iso-STAT could cause Ebi3 expression to produce IL-35^+^ cells such as that binding of phosphorylated STAT3 with STAT1 can induce L-35^+^ T cells.^46,47^ When GPRs are activated by ligands such as CLA, the α subunits of heterotrimeric G proteins coupled to the receptor will disassociate from the βγ subunits. This can further affect intracellular signaling protein or directly target functional proteins depending on the type of α subunits such as Gα_s_, Gα_i/o_, Gα_q/11_, and Gα_12/13_.^67^ We found that G protein Gα_q/11_ is involved in the CLA mediated IL-35 expression. G protein α chain isoform Gα_q/11_ could activate JAK1/2 to induce activation of STAT1 and STAT4. Others also found that the combination of STAT1/4 could cause the alteration of M1 macrophages to M2 macrophages.^68^ Gαi-mediated TRPC4 activation by polycystin-1 contributes to the endothelial function via STAT1 activation.^69^ Previous findings also indicated that the combination of STAT1 and STAT4 could cause iTr35 Treg cells. While the mice were treated with IL-35, STAT1 and STAT4 were phosphorylated to expand Treg cells.^49^ Our data show that CLA induced the activation of JAK1/2 and STAT1/4 to promote the expression of *Ebi3* and *Il12a*, causing the generation of IL-35 in macrophages. However, it is not clear why STAT1/STAT4 heterodimers can bind to *Ebi3* and *Il12a* promoters to transcribe *Ebi3* and *Il12a* in the macrophages, while STAT1 and STAT4 homodimers do not.^70^

Our results show that CLA can induce expression of IL-35 in macrophages to maintain gut homeostasis. These data are consistent with previous results that IL-35 recombinant protein can reverse IBD and psoriasis through regulation of inflammatory cytokines and immune cells.^40^ IL-35 can influence on the occurrence and development of colonic inflammation. There was a negative correlation between serum IL-35 levels and Mayo score in ulcerative colitis (UC) patients.^71^ *Ebi3*-/- and *p35*-/- mice were more sensitivity to colitis.^29,30^ Previous studies showed that CLA produced by locally probiotic bacteria in the gut that targets macrophage PPARγ to suppress colitis.^72^

In conclusion, we here found that REG 4/Reg4 associated *Lactobacillus*, which can metabolize LA to CLA, maintains gut immune homeostasis through inducing IL-35 production in the macrophages of colon tissues. Although this study is limited to animals, our findings should potentially offer the means for the prevention and therapy of inflammation-associated gut diseases.

## Methods and materials

### Reagents

Reagents and oligoes used in the study were listed in Table S1.

### Mice

Total 200 four- to six-week-old male or female C57BL/6 mice were offered by Nanjing Animal Center, Nanjing, China. Human REG4 transgenic mice (*huREG4*^*IECtg*^) were generated in Nanjing Animal Center as previously described.^39,73^ Reg4 deficient (KO) mice were prepared.^37^ All experimental procedures were done according to the Institutional Animal Care and Use Committee of the Model Animal Research Center. Institute’s Animal Ethical Committee of Nankai University approved all animal experiments (Ethical number: ECNK20170802). Mice were kept in a specific-pathogen free (SPF) facility (Animal center, Nankai university) on a 12-h light/dark cycle and provided with food and water. These mice were demonstrated to be free of disease-causing pathogens that could affect mouse health. Unless otherwise noted, mice were housed together, with six mice per cage. Age- and gender-matched mice were used in the experiments. Experimental variables such as husbandry, environmental influences and parental genotypes were controlled. C57BL/6 germ-free (GF) mice were prepared by the Beijing Animal Center. Experiments on GF mice were made in Institute of Laboratory Animal Science, Chinese Academy of Medical Sciences (CAMS) Comparative Medical Center.

### Mouse models

Dextran sodium sulfate (DSS) mediated colitis was done according to our previously reported method.^74^ Briefly, six mice per group (experimental and control groups) were exposed to DSS (2.5%) in their drinking water for 7 d at the indicated dose and then started to regular drinking water. For survival studies, mice were observed after DSS treatment for 12 d. Mice were weighed every other day for the determination of percent weight change. % weight change = (weight at day X-day 0/weight at day 0) × 100 was used to calculate percent weight change. Rectal bleeding, diarrhea, and general signs of morbidity such as hunched posture and failure to groom were also monitored. Disease activity index (DAI) and histological evaluation were assessed according to previous methods.^37,75^

Gut microbiota transplantation was performed according to the previous method.^39^ Mice were treated with pan-antibiotics (ampicillin (A, 1 g/L, Sigma), vancomycin (V, 0.5 g/L, Sigma), neomycin sulfate (N, 1 g/L, Sigma), and metronidazole (M, 1 g/L, Sigma) in their drinking water for 2 weeks. The drinking water containing the antibiotics was exchanged per 3 d. The stools from antibiotic-treated mice were collected and cultured under anaerobic and aerobic conditions to confirm the elimination of bacteria. Then, mice were orally administered 200 μL of the fecal suspension or bacteria using glycerol stocks and used for other experiments after 3 d. The cecal contents from detergent-treated mice or 1 × 10^9^ CFU were suspended in 1 mL phosphate buffered saline (PBS) with 30% glycerol.

For conjugated linoleic acid (CLA) of germ-free (GF) mice, GF mice were treated by CLA gavage (100 g/kg).

For the administration of neutralizing antibody and cytokine on mice, 100 μL volumes of anti-IL-35 blocking antibody (100 μg/mL, Sigma) or rIL-35 (2.5 μg/mL, PEPROTECH) suspended in PBS were injected subcutaneously (twice, one dose per 3 d). Mice injected with 100 μL PBS were used as control mice.

For transplant experiment of bone marrow cell (BMC), recipient mice were irradiated using a Shepherd Mark I Cesium Irradiator (J.L. Shepherd and Associates) (800 Gy, a single dose). The BMCs collected from donor mice were injected into irradiated recipient mice (2 × 10^6^ cells per mouse) via the tail vein.

### In vitro macrophage generation

For bone marrow-derived macrophages (BMDMs), mouse bone-marrow cells were obtained as described previously.^76,77^ Briefly, mouse bone-marrow cells were collected by removing the femurs of mice, and flushing out the marrow in DMEM medium. The red blood cells were lysed with an ammonium chloride buffer and then plated into a 75-cm^2^ flask in medium. The medium was supplemented with 20ng/ml murine M-CSF cytokine in the presence of 10% FCS and 1% penicillin and streptomycin. The medium was changed every 2 d, and after cultured for 7 d, cells were harvested and then divided into 24-well plates for further *Ex vivo* stimulation.

For human peripheral blood monocyte cells derived macrophages (BMDMs), fresh whole blood was collected from healthy volunteers presented in Tianjin Blood Center (Tianjin), which was approved by Blood Center in Tianjin. Human PBMCs were isolated by density centrifugation on the day of blood collection using Ficoll-Paque medium (Solarbio) according to manufacturer’s instructions. PBMCs were cultured in the presence of M-CSF (500 U/mL) for 5 d for further *ex vivo* stimulation.

### Ex vivo stimulation

For the co-culture of macrophages, B and T cells, the immune cells from spleen by were stimulated with CLA (50 μM）with or without anti-IL-35 blocking antibody for 24 h. For sorted F4/80^+^ macrophages, CD19^+^ B cells and CD4^+^ T cells, F4/80^+^ macrophages, CD19^+^ B cells and CD4^+^ T cells first were sorted from spleen by flow cytometry, and then stimulated by CLA for 24 h. After co-cultured for 24 h, cells were collected for flow cytometry analyses.

For the immune cells treated by different kinds of inhibitors, 5 × 10^6^ macrophages per well were seeded into 24-wells plate. Cells were pre-incubated with (Experimental groups) or without (Control groups) GPR40 inhibitor (DC260126, 10 μM), GPR120 inhibitor (AH-7614, 10 μM), STAT1 inhibitor (Fludarabine, 10 μM), STAT4 inhibitor (Cinnamon bark, 10 μM), Gα/q inhibitor (YM-254890, 10 μM), Gαi/o inhibitor rimonabant (10 μM) and Gβ and γ-chain inhibitor gallelin (10 μM) for 30 min, and then cells were stimulated with linoleic acid (LA) (50μM), conjugated linoleic acid (CLA, 50 μM), cis-9 CLA isomers (c9t11, 50 μM), trans-10 CLA isomers (t10c12, 50 μM) or LPS (100 ng/mL) or adenylsuccinic acid (ALA, 50μM) for 24 h. Cells were collected for flow cytometry analyses. These treatments could determine the CLA-mediated signal pathway(s).

For the cells treated by small interfering RNA (siRNA), the macrophages were seeded in six-well plates overnight to reach approximately 50% confluence. Oligonucleotides were purchased from Sangon (BGI, Beijing). The sequences of the siRNAs of murine and human STAT1 and STAT4 were listed in Table S1. Transfections were performed using Lipofectamin 3000 according to manufacturer’s instructions (Invitrogen) for 24 hr at a final oligonucleotide concentration of 10 nM and then stimulated using CLA for 24 h.

### Sequencing of gut microbiota

The sequencing of gut microbiota was performed based on our previously reported method.^78^ Briefly, using primers that target to the V3–V4 regions of the 16S rRNA, gut microbiota was analyzed by Majorbio Biotechnology Company (Shanghai, China). After PCR was completed, the amplicons were purified, quantified, normalized, and pooled, and then followed by sequencing using Titanium chemistry (Roche, Basel Switzerland) according to the manufacturer’s protocol. Operational Taxonomic Unit (OTU) analysis was performed according to the previous method.^78^

### Isolation of Lactobacillus

Bacterium was isolated according to previous protocol.^79^ Rogosa SL selective medium (Sigma-Aldrich) was used to isolate *Lactobacillus* from fresh stool samples from *huREG4*^*IECtg*^ mice. The colonies were identified and purified through 16s ribosomal DNA sequence analyses. The isolated *Lactobacillus* was cultured in MRS (deMan-Rogosa-Sharpe) media and also grown on MRS agar containing 10% sucrose. The sachets of AnaeroPack-Anaero (Mitsubishi Gas Chemical, Japan) were used to generate anaerobic conditions in an air-tight jar.

For screening CLA-producing *Lactobacillus* strain(s), the previous procedures^80^ were used in this study. The lactobacilli were incubated anaerobically in MRS broth containing linoleic acid (0.5 mg/mL) and 2% (wt/vol) Tween 80 at 37°C for 24 h.

After incubation, the culture was centrifuged at 20,800 × g for 1 min, collected the supernatant, and then mixed with 1 mL of isopropanol by vortexing. The fatty acids were extracted by vortexing the solution and then mixed with 1.5 mL of hexane. The CLA in the culture supernatant was analyzed using a spectrophotometer (smart plus SP-1900PC) at 233 nm.

### Cell isolation and flow cytometry

Previous protocol was used in cell isolation and flow cytometry.^74^ The colons were isolated from mice and cleaned by shaking in ice-cold PBS. The lamina propria (LP) tissues were incubated in digestion buffer (DMEM, 5% fetal bovine serum, 1 mg/mL Collagenase IV (Sigma-Aldrich) and DNase I (Sigma-Aldrich) for 40 min. Then, lamina propria (LP) lymphocytes were isolated using Percoll gradient separation by centrifugation for 20 min at 1,800 rpm at room temperature (26 °C) and collected at the interphase of the Percoll gradient, washed and then stained and analyzed by flow cytometry. After 7-AAD staining, dead cells were eliminated. For sorting macrophage, B and T cells, the spleen cells from mice were stained using F4/80 antibody, CD19 antibody and CD4 antibody, respectively, and then sorted using flow cytometry.

For intracellular staining, the isolated cells were cultured and stimulated with phorbol 12-myristate 13-acetate (PMA, 50 ng/mL, Sigma) and ionomycin (1 μg/mL, Sigma) in the presence of GolgiStop (10 ng/mL, BD Biosciences). After 6 h, cells were washed in PBS, fixed in Cytofix/Cytoperm, permeabilized with Perm/Wash buffer (BD Biosciences), and then stained with different primary antibodies. Meanwhile, dead cells were eliminated after 7-AAD staining.

### LC-MS (liquid chromatography-mass spectrometry)

Liquid chromatography-mass spectrometry (LC-MS) was performed according to our previously reported method.^39^ Briefly, all samples were extracted with methanol, and an internal standard (DL-O-chlorophenylalanine) was added. The samples were analyzed by ACQUITYTM UPLC-QTOF. The data were preprocessed with Masslynx 4.1 software (Waters) and then edited into a two-dimensional data matrix by Excel 2010 software and then analyzed using SIMCA-P 13.0 software (Umetrics AB, Umea, Sweden).

### Immunoblot, immunoprecipitation, H & E staining, immunostaining, and ELISA

Immunoblot and immunoprecipitation which were used to determine CLA mediated signaling, hematoxylin/eosin (H & E) staining and immunostaining, which were used to investigate inflammation, and enzyme linked immunosorbent assay (ELISA), were performed according to our previously reported methods.^39,81^

### Statistical analysis

The statistical significance of the survival curves was estimated using Kaplan and Meier method, and the curves were compared using the generalized Wilcoxon’s test. Histological scores were analyzed using a Mann-Whitney U test, which was used to compare differences between two independent samples or populations. ONE-way ANOVA Bonferroni’s Multiple Comparison Test and two side Student’s t-test, which were mainly used for normally distributed data, were used. A 95% confidence interval was considered significant and defined as *p* < 0.05. *, *p* < 0.05; **, *p* < 0.01; ***, *p* < 0.001.

## Supplementary Material

Supplementary data.docx

## Data Availability

RAW16s rRNA gene sequence data: BioProject, PRJNA695415; http://www.ncbi.nlm.nih.gov/bioproject/695415.
