# Supplementary material for: Gut microbiota CLA and IL-35 induction in macrophages through Gαq/11-mediated STAT1/4 pathway: an animal-based study
Source: Gut Microbes. 2024 Dec 5;16(1):2437253. doi: 10.1080/19490976.2024.2437253 (PMC11622586; doi:10.1080/19490976.2024.2437253)
Supplement: Supplementary data.docx [file KGMI_A_2437253_SM0886.docx]

**Gut microbiota CLA and IL-35 induction in macrophages through Gαq/11-mediated STAT1/4 pathway: An animal-based study**

Xiaomin Su^1^, Yazheng Yang^1^, Yunhuan Gao^1^, Juanjuan Wang^1^, Yang Hao^1^, Yuan Zhang^1^ & Rongcun Yang^1,2,3^

^1^Department of Immunology, Nankai University School of Medicine, Nankai University, Tianjin 300071, China;

^2^Translational Medicine Institute, Tianjin Union Medical Center of Nankai University, Tianjin, 300121, China;

^3^State Key Laboratory of Medicinal Chemical Biology, Nankai University, Tianjin 300071, China;


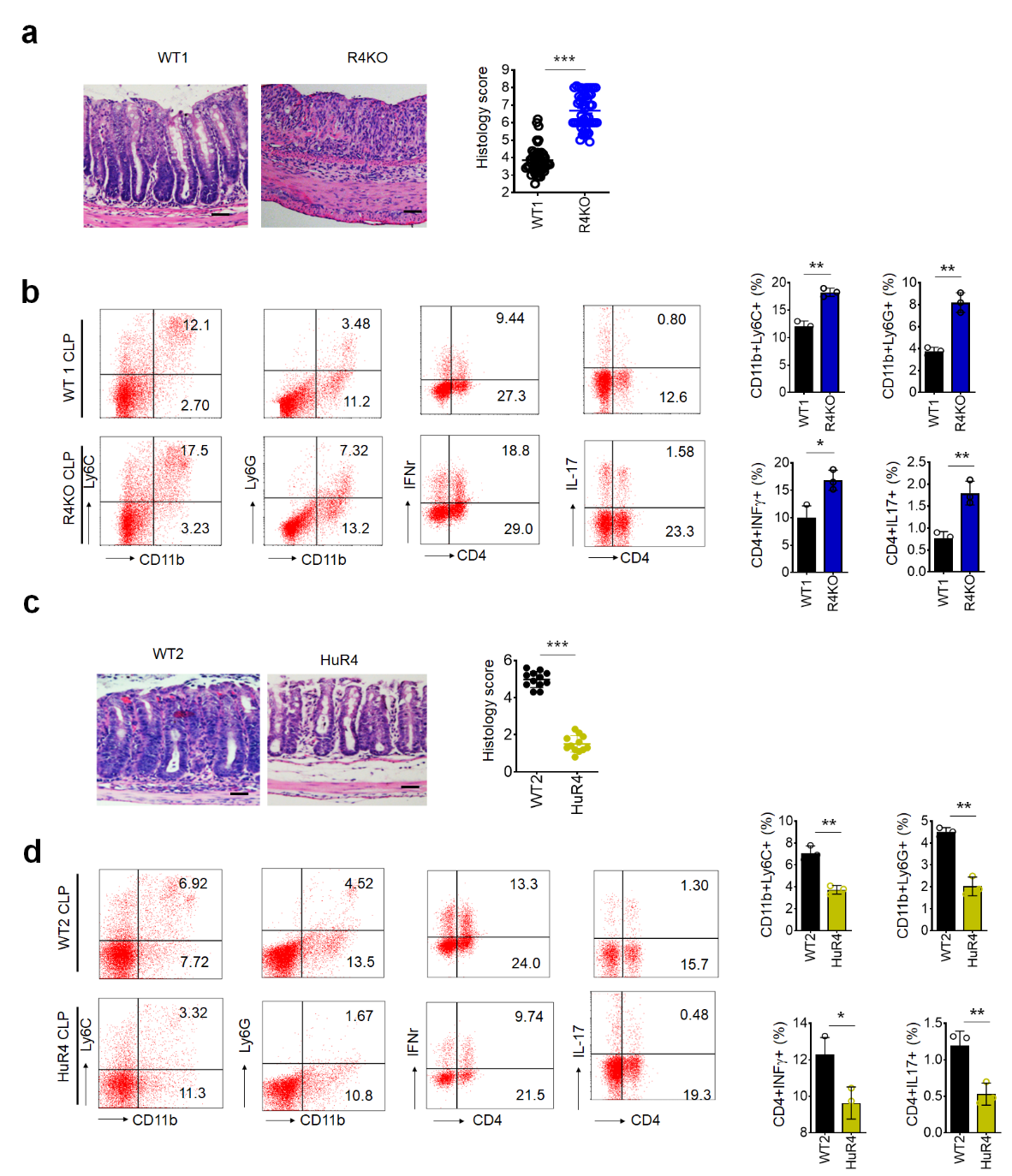


**Figure S1.** Reg4/huREG4 resists to DSS-mediated colitis. a) H&E staining of colon tissues of *Reg4* KO (R4KO) and wild type (WT1) mice after giving 2.5% DSS for 7 days. b) Flow cytometry of CD11b^+^Ly6c^+^, CD11b^+^Ly6G^+^, CD4^+^IFNγ^+^ and CD4^+^IL-17^+^ cells in colon lamina propria (CLP) of R4KO and WT1 mice after giving 2.5% DSS for 7 days. Isotypic Ctr, Isotypic control; c) H&E staining of colon tissues of WT1 and *huREG4^IECtg^* (huR4) mice after giving 2.5% DSS for 7 days. d)Flow cytometry of CD11b^+^Ly6c^+^, CD11b^+^Ly6G^+^, CD4^+^IFNγ^+^ and CD4^+^IL-17^+^ cells in CLP of WT2 and HuR4 mice after giving 2.5% DSS for 7 days. Student’s *t*-test. **p<0.05,* ***p<0.05,* ****p<0.05.* Data is a representative of at least two experiments.

**
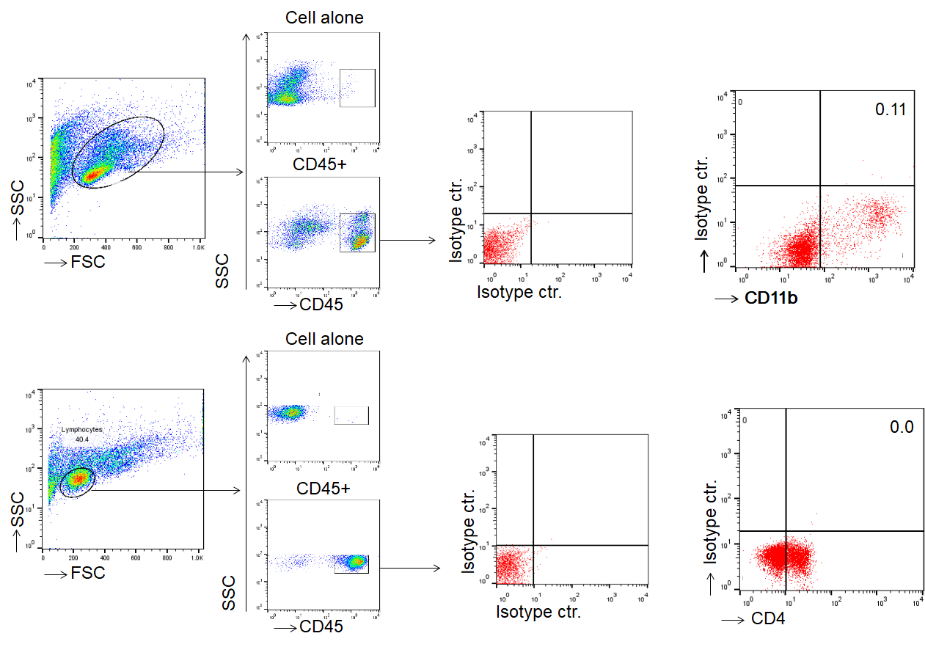
**

**Figure S2.** Gating strategies for the flow cytometry in supplementary Figure S1.

Isotypic ctr., isotypic control.


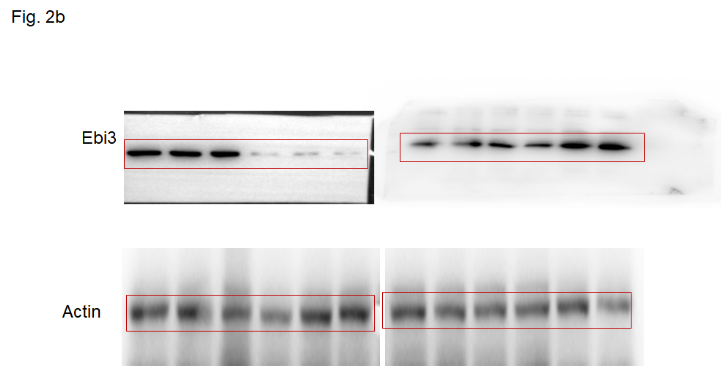


**Figure S3.** Raw data for figure 2b.


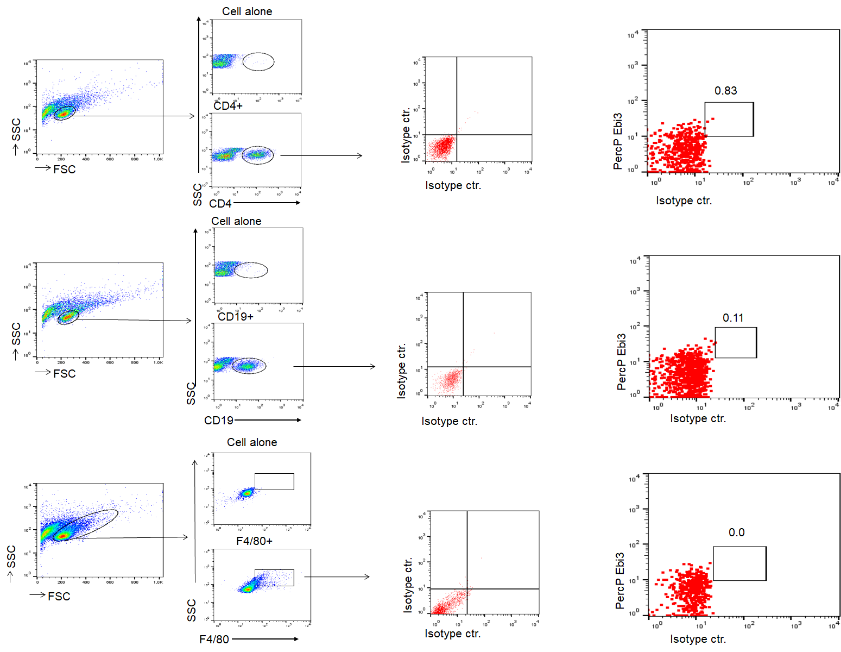


**Figure S4.** Gating strategies for flow cytometry and isotypic control (ctr) in Figure 2.


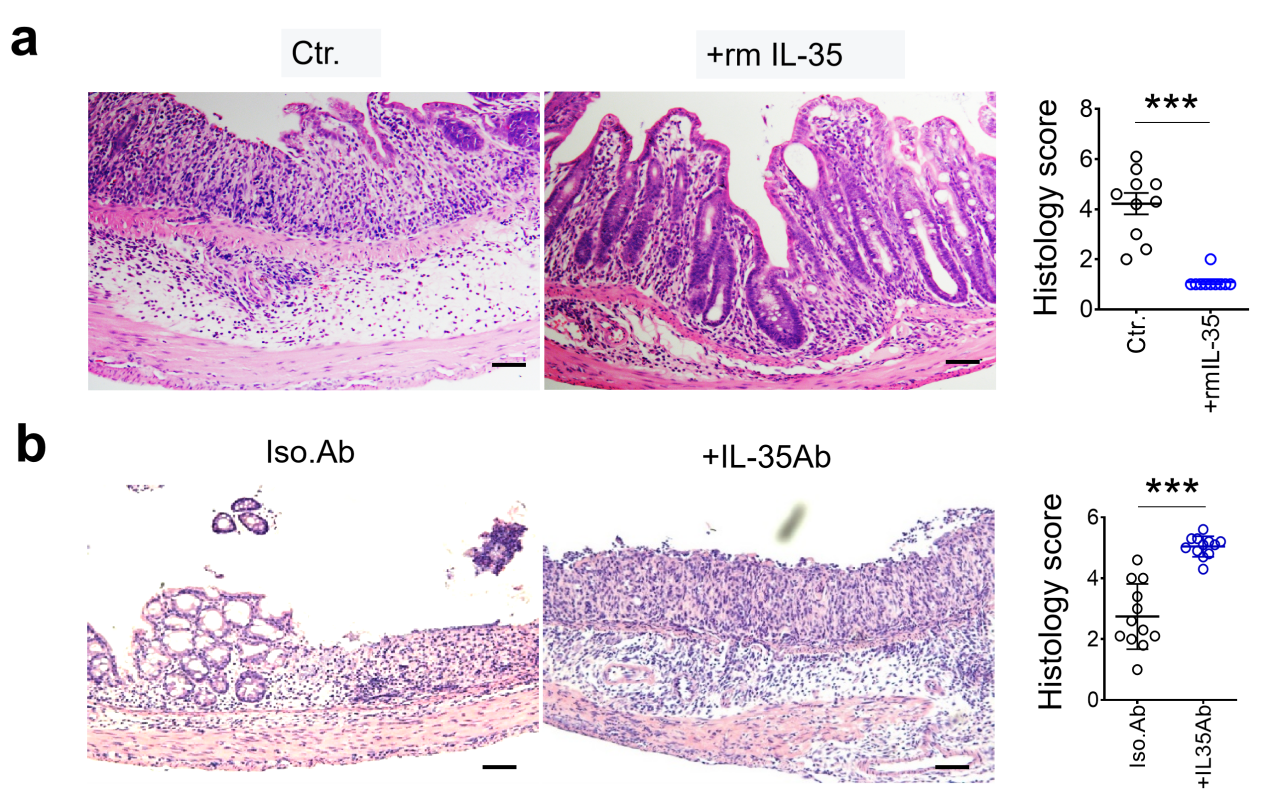


**Figure S5.** REG4/Reg4-mediated resistance to DSS-induced colitis depends on IL-35. a) H&E staining of colon tissues of Reg4 KO mice with (+rmIL-35) or without (Ctr.) rmIL-35 after giving 2.5% DSS for 7 days. b) H&E staining of *huREG4^IECtg^* mice with (+IL-35 Ab) or without (isotypic control, iso) IL-35blocking antibody injection after giving 2.5% DSS for 7 days. Student’s *t*-test. Data is a representative of at least two experiments.


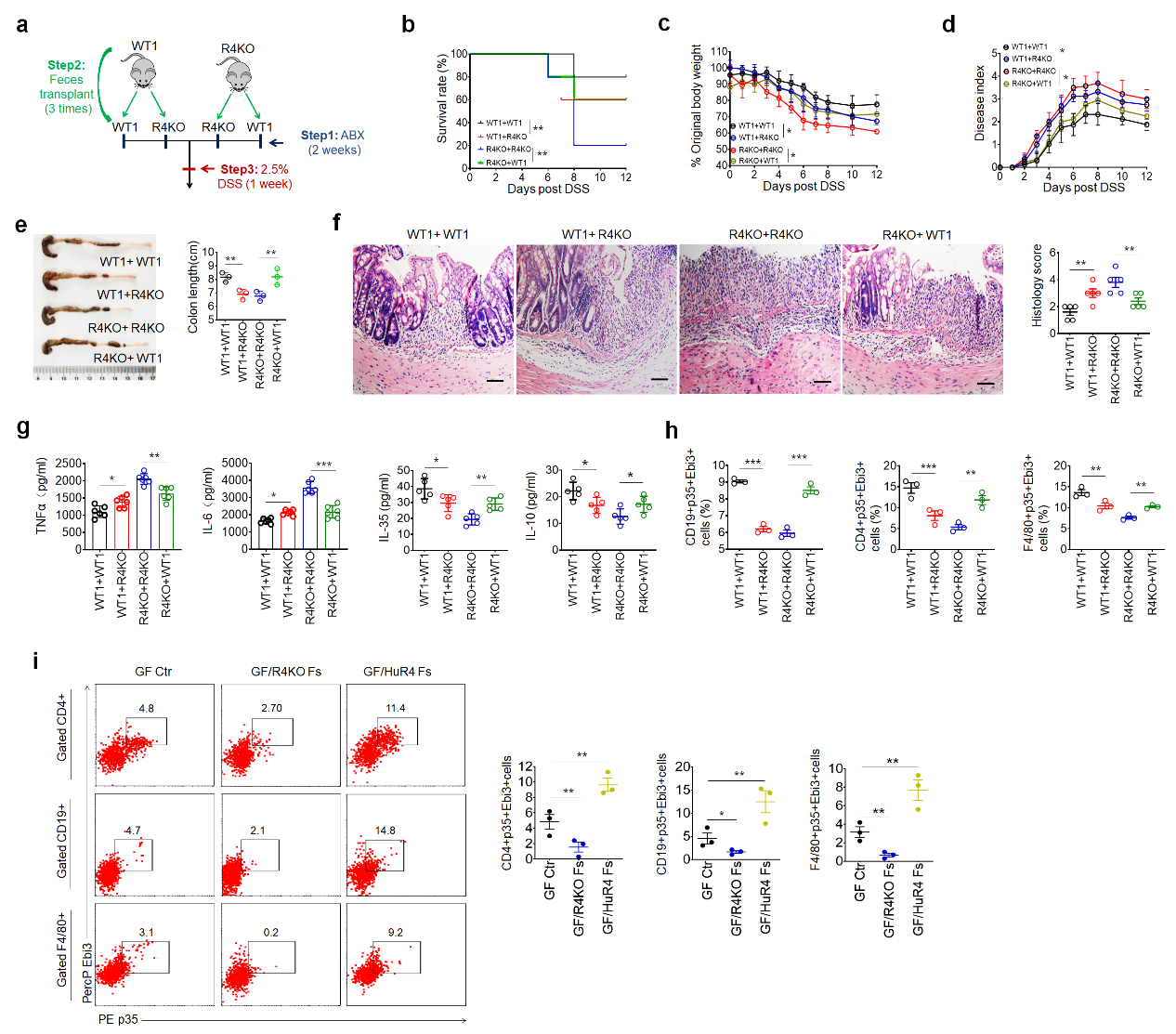


**Figure S6.** REG4/Reg4 mediated resistance to DSS induced colitis depends on gut microbiota. a) A schematic illustration showing the design for feces transplantation studies. b) Survival rate; c) Body weight changes; d) Disease activity index; e) Colon length; f) H&E staining; g) ELISA of TNFα, IL-6, IL-35 and IL-10 in sera; h) Flow cytometry of CD4^+^p35^+^Ebi3^+^, CD19^+^p35^+^Ebi3^+^ and F4/80^+^p35^+^Ebi3^+^ cells in colon lamina propria (CLP); Mice transplanted using the feces of R4KO or WT1 mice were given 2.5% DSS for 7 days. WT1+WT1, the feces of WT1 into WT1 mice; WT1+R4KO, the feces of WT1 into R4KO mice; R4KO+R4KO, the feces of R4KO into R4KO mice; R4KO+WT1, the feces of R4KO into WT1 mice

i) Flow cytometry of CD4^+^p35^+^Ebi3^+^, CD19^+^p35^+^Ebi3^+^and F4/80^+^p35^+^Ebi3^+^ cells in CLP tissues of germ free (GF)mice transplanted using the feces of R4KO (GF/R4KOFs）or with feces of HuR4 (GF/HuR4 Fs). Wilcoxon’s test in b; One-way ANOVA test in c-i; **p<0.05,* ***p<0.05,* ****p<0.05.* Data is a representative of at least two experiments.


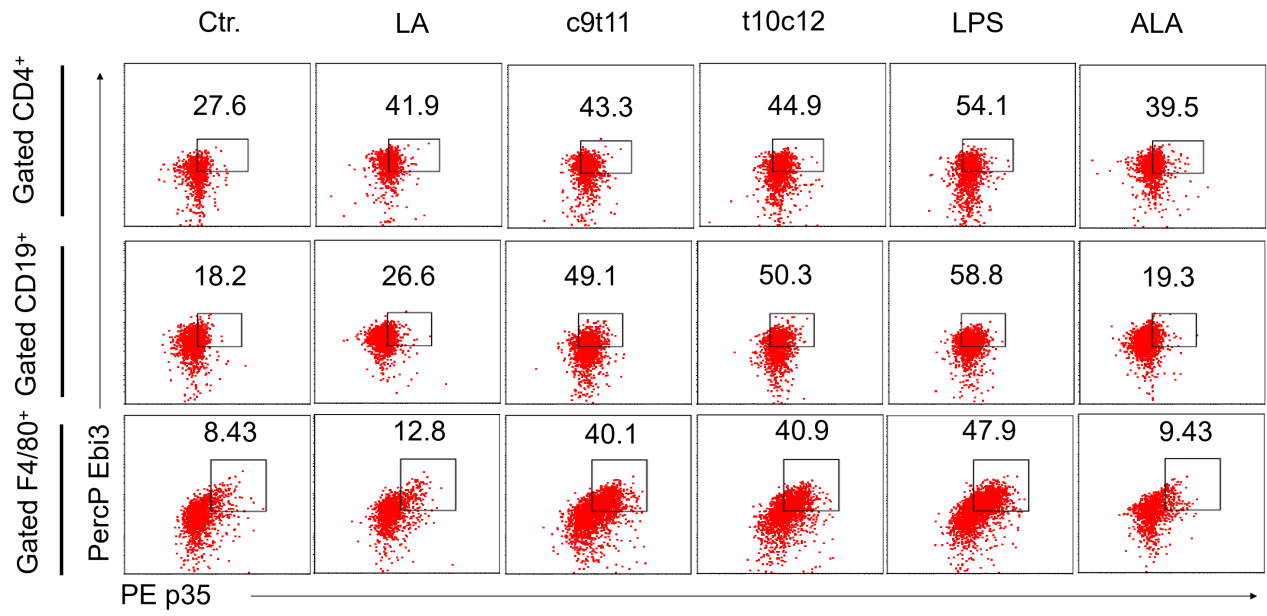


**Figure S7.** CLAs induces IL-35 cells in vitro co-cluture of macrophages with T and B cells. Flow cytometry of CD4^+^p35^+^Ebi3^+^, CD19^+^p35^+^Ebi3^+^and F4/80^+^p35^+^Ebi3^+^ cells in vitro coculture of immune cells after exposure to CLA (c9t11 or t10c12), ALA or LPS, Ctr.: no stimulation control.

**
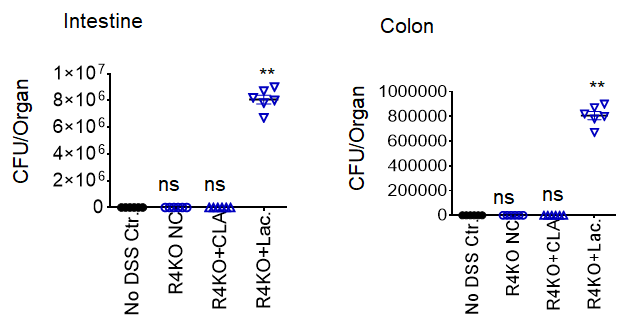
**

**Figure S8.** *Lactobacillus* colonization in intestine and colon. Mice were treated with pan-antibiotics in their drinking water for two weeks, and then 200 μL(1×10^9^ CFU *Lactobacillus* /ml) were infused into mice after 3 days. After infusing into mice for 7 days, CFU in intestine and colon were culture in *Lactobacillus* medium. 100% CFU was infused *Lactobacillus Reuteri* through 16s ribosomal DNA sequence analyses. Student’s *t*-test. Ns, no significance.

*
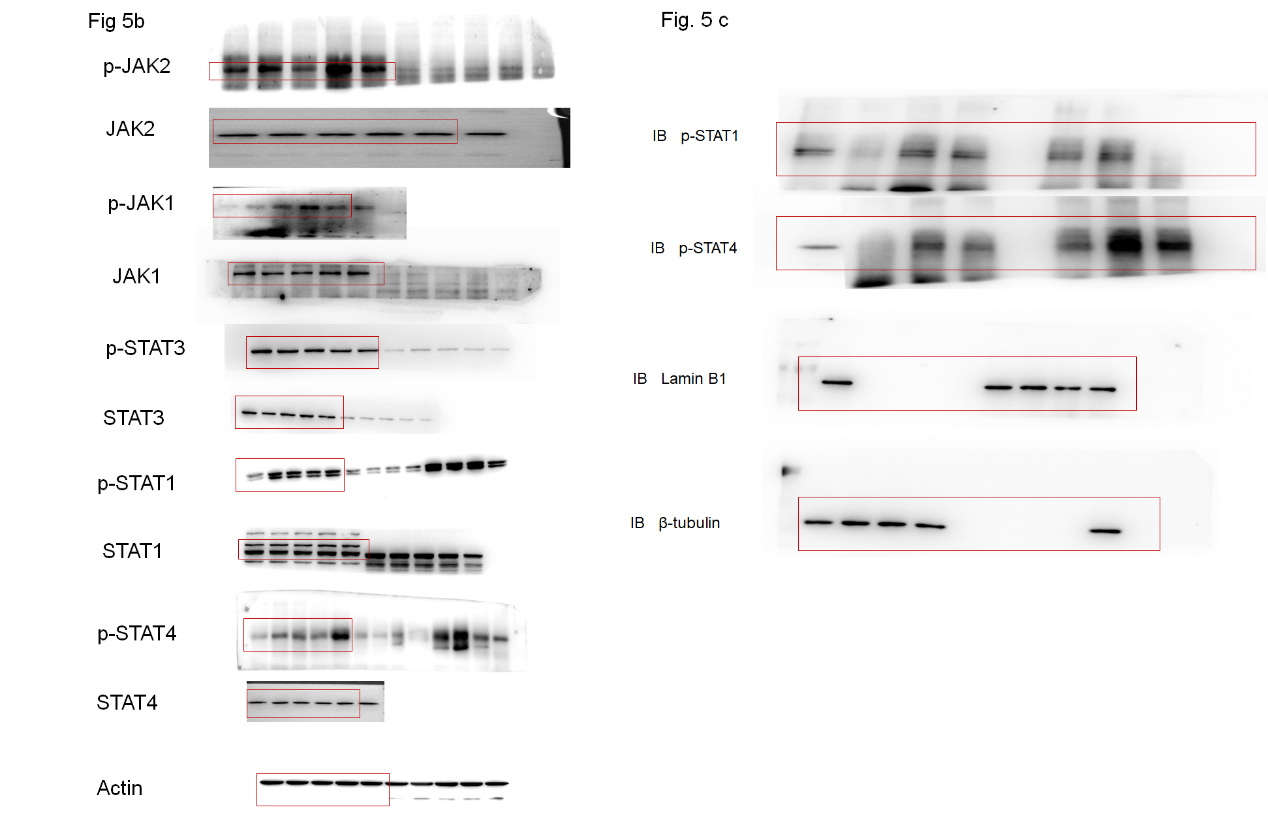
*

**Figure S9.** Raw data for Figure 5 b and c.


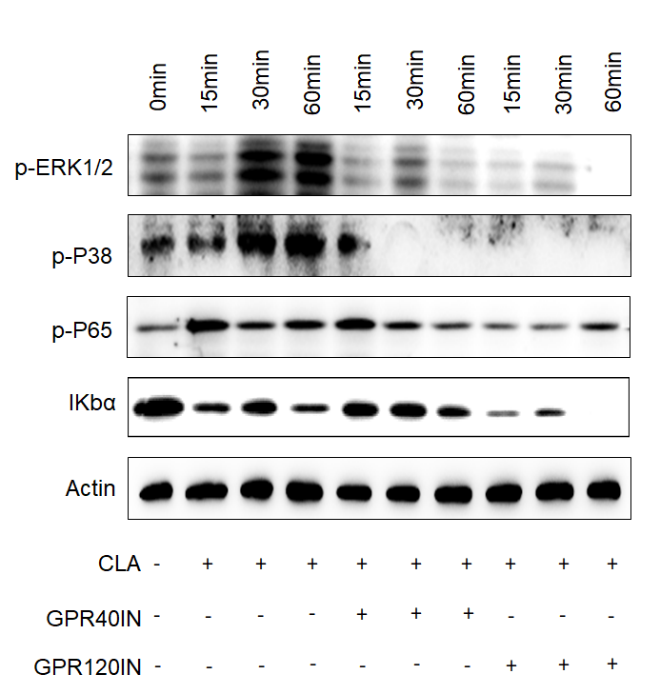


**Figure S10.** CLA induces the activation of ERK1/2, P38，P65 and IκBα in the BMDMs. Immunoblotting of phospho-ERK1/2/P38/P65 and IκBα in the bone marrow derived macrophages (BMDMs) upon exposure to CLA with or without GPR40IN, GPRR120IN inhibitor at the indicated time. Actin as the loading control.


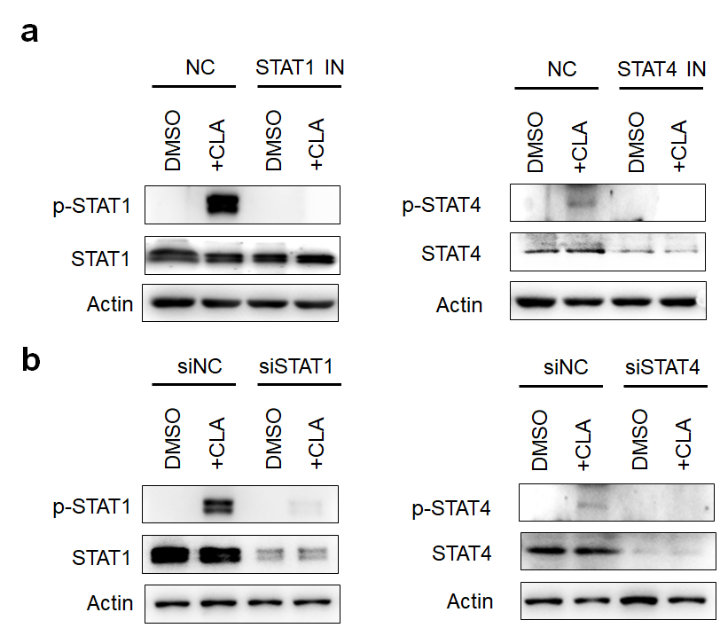


**Figure S11.** STAT1 or STAT4 inhibitor or their siRNA affects CLA mediated phosphorylation of mouse STAT 1 or STAT4. Immunoblotting of phospho-STAT1(a) and STAT4 (b), and total STAT1 and STAT4 in BMDM upon exposure to CLA in the presence of STAT1 (STAT1 IN) or STAT4 (STAT4 IN) inhibitors, or STAT1 (siSTAT1) or STAT4 siRNA (siSTAT4).


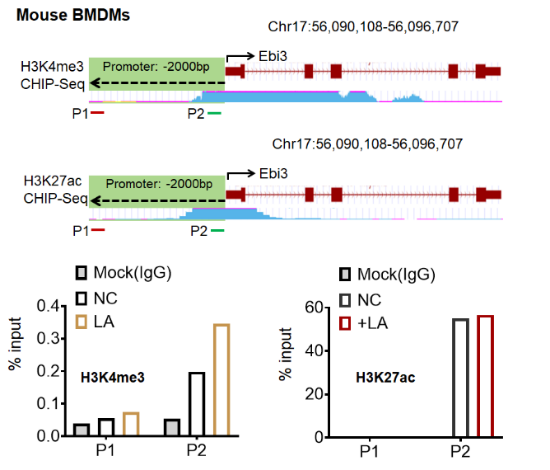


**Figure S12.** CLA induces enrichment of H3K4me3 in the promoter region of IL-35 subunit Epi3. ChIP-PCR of H3K4me3 and H3K27ac modification on the promoter region of Ebi3 in the BMDMs upon exposure to CLA for 24hrs.NC, unstimulated control. One-way ANOVA test. Data is a representative of at least two experiments.


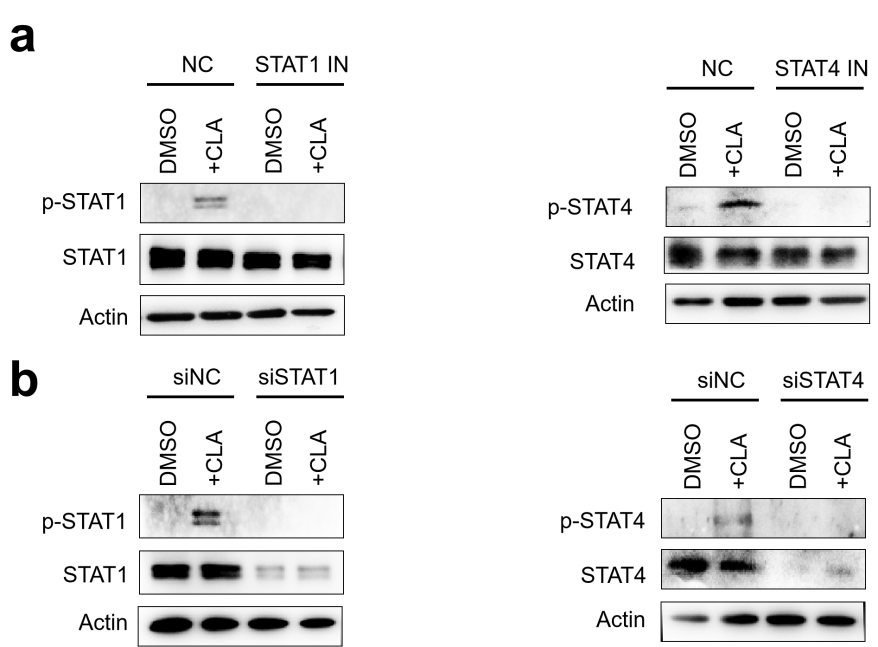


**Figure S13.** STAT1 or STAT4 inhibitor (a) or siRNA (b) affects CLA mediated phosphorylation of STAT 1 or STAT4 in human monocytes derived macrophages. Immunoblotting of phospho-STAT1 and STAT4, and their total STAT1 and STAT4 in the human peripheral blood monocyte cells derived macrophages upon exposure to CLA in the presence of STAT1 or STAT4 inhibitors, or STAT1 and STAT4 siRNA.


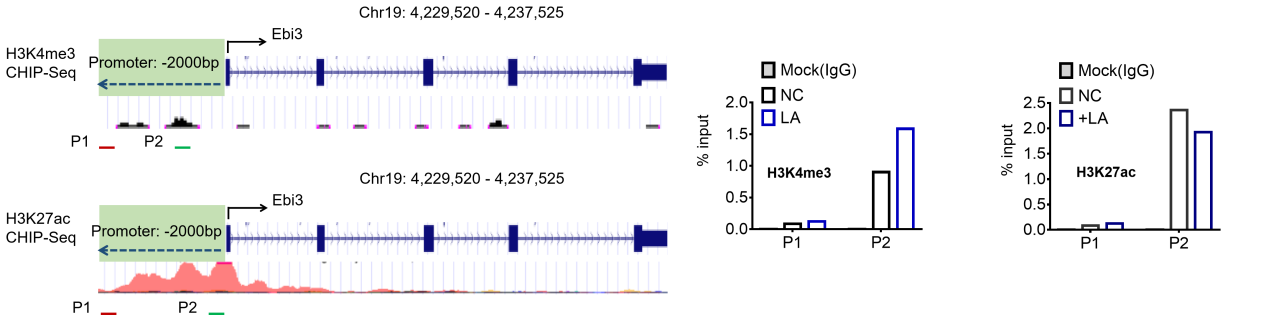


**Figure S14.** CLA induces enrichment of H3K4me3 in the promoter region of IL-35 subunit Epi3. ChIP-PCR of H3K4me3 and H3K27ac modification on the promoter region of Ebi3 in the human peripheral blood monocyte cells derived macrophages upon exposure to CLA for 24hrs. NC, unstimulated control. One-way ANOVA test. Data is a representative of at least two experiments.

**Table S1. Reagents and oligoes used in this study.**

| REAGENT or RESOURCE | SOURCE | IDENTIFIER |  |
| --- | --- | --- | --- |
| Antibodies for Western blot | | | |
| Anti-STAT1 Antibody | Proteintech | Cat: 10144-2-AP RRID:AB_2286875 |  |
| Anti-STAT3 Antibody | Cell Signaling Technology | Cat: 9139 RRID:AB_331757 |  |
| Anti-STAT4 Antibody | Proteintech | Cat: 13028-1-AP RRID:AB_2196604 |  |
| Anti-JAK1 Antibody | BD Biosciences | Cat: 610232 RRID:AB_397627 |  |
| Anti-JAK2 Antibody | Abcam | Cat: ab108596 AB_10865183 |  |
| Anti-P65 Antibody | Abcam | Cat: ab32536 RRID:AB_776751 |  |
| Anti-ERK1/2 Antibody | Abcam | Cat: ab184699 RRID:AB_2802136 |  |
| Anti-P38 Antibody | Proteintech | Cat: 14064-1-AP RRID:AB_2878007 |  |
| Anti-JNK Antibody | Proteintech | Cat: 66210-1-Ig RRID:AB_2881601 |  |
| Anti-IκBα Antibody | Abcam | Cat: ab32518 RRID:AB_733068 |  |
| Anti-Ebi3 Antibody | Abcam | Cat: ab124694 RRID:AB_10972489 |  |
| Anti-β-Actin Antibody | Santa Cruz | Cat: sc-47778 RRID:AB_626632 |  |
| Anti-Lamin B1 Antibody | Abcam | Cat: ab16048 RRID:AB_443298 |  |
| Anti-βTubulin Antibody | Abcam | Cat: ab6046 RRID:AB_2210370 |  |
| Anti-p-STAT1 Antibody | Affinity | Cat: AF3300 RRID:AB_2834719 |  |
| Anti-p-STAT3 Antibody | ABclonal | Cat: AP0474 RRID:AB_2771567 |  |
| Anti-p-STAT4 Antibody | Affinity | Cat: AF3441 RRID:AB_2834883 |  |
| Anti-p-JAK1 Antibody | ABclonal | Cat: AP0530 AB_2771224 |  |
| Anti-p-JAK2 Antibody | ABclonal | Cat: AP0531 RRID:AB_2771226 |  |
| Anti-p-P65 Antibody | Cell Signaling Technology | Cat: 3037 RRID:AB_2341216 |  |
| Anti-p-ERK1/2 Antibody | Proteintech | Cat: 28733-1-AP RRID:AB_2881202 |  |
| Anti-p-P38 Antibody | Proteintech | Cat: 28796-1-AP RRID:AB_2918205 |  |
| Anti-p-JNK Antibody | Proteintech | Cat: 80024-1-RR RRID:AB_2882943 |  |
| Goat anti-mouse IgG (H+L) -HRP antibody | ABclonal | Cat: AS003 RRID:AB_2769851 |  |
| Goat anti-rabbit IgG (H+L) -HRP antibody | ABclonal | Cat: AS014 RRID:AB_2769854 |  |
| Goat anti-mouse IgG (L) -HRP antibody | ABclonal | Cat: AS062 RRID:AB_2864056 |  |
| mouse anti-rabbit IgG (L) -HRP antibody | ABclonal | Cat: AS061 RRID:AB_2864055 |  |
| Antibodies for flow cytometry analysis |  | | |
| FITC anti-mouse CD19 Antibody | Biolegend | Cat: 152404 RRID:AB_2629813 |  |
| FITC anti-mouse CD4 Antibody | Biolegend | Cat: 100406 RRID:AB_312691 |  |
| FITC anti-mouse F4/80 Antibody | Biolegend | Cat: 123108 RRID:AB_893502 |  |
| PE anti-mouse/human p35 Antibody | R&D | Cat: IC2191P RRID:AB_1964620 |  |
| PerCP anti-mouse Ebi3 Antibody | R&D | Cat: IC18341C RRID:AB_2097431 |  |
| FITC anti-human CD19 Antibody | Biolegend | Cat: 302205 RRID:AB_314235 |  |
| FITC anti-human CD4 Antibody | Biolegend | Cat: 300505 RRID:AB_314073 |  |
| FITC anti-human CD14 Antibody | Biolegend | Cat: 325603 RRID:AB_830676 |  |
| PE anti-human Ebi3 Antibody | Biolegend | Cat: 360903 RRID:AB_2562879 |  |
| Apc anti-human p35 Antibody | R&D | Cat: IC2191A RRID:AB_1964616 |  |
| FITC anti-mouse/human CD11b Antibody | Biolegend | Cat: 101206 RRID:AB_312789 |  |
| PE anti-mouse Ly6g Antibody | Biolegend | Cat: 127607 RRID:AB_1186104 |  |
| PE anti-mouse Ly6c Antibody | Biolegend | Cat: 128007 RRID:AB_1186133 |  |
| PE anti-mouse IFNγ Antibody | Biolegend | Cat: 505808 RRID:AB_315402 |  |
| PE anti-mouse IL-17 Antibody | Biolegend | Cat: 506903 RRID:AB_315463 |  |
| Antibodies for Immunofluorescence analysis |  | | |
| Anti-mouse/human-Ebi3 Antibody | Proteintech | Cat: 12371-1-AP RRID:AB_10646470 |  |
| Anti-mouse CD4 Antibody | Proteintech | Cat: 67786-1-Ig RRID:AB_2918550 |  |
| Anti-mouse CD19 Antibody | Proteintech | Cat: 27949-1-AP RRID:AB_2881019 |  |
| Anti-p--STAT1 Antibody | Affinity | Cat: AF3300 RRID:AB_2834719 |  |
| Anti-p-STAT4 Antibody | Santa cruz | Cat: sc-28296 RRID:AB_628294 |  |
| DAPI | Thermo Fisher Scientific | Cat: D3571 RRID:AB_2307445 |  |
| CoraLite594 conjugated Goat Anti-Mouse IgG(H+L) Antibody | Proteintech | Cat: SA00013-3 RRID:AB_2797133 |  |
| CoraLite594 conjugated Goat Anti-Rabbit IgG(H+L) Antibody | Proteintech | Cat: SA00013-4 RRID:AB_2810984 |  |
| CoraLite488 conjugated Goat Anti-Mouse IgG(H+L) Antibody | Proteintech | Cat: SA00013-1 RRID:AB_2810983 |  |
| CoraLite488 conjugated Goat Anti-Rabbit IgG(H+L) Antibody | Proteintech | Cat: SA00013-2 RRID:AB_2797132 |  |
| ELISA kits |  |  |  |
| Mouse IL-35 | mlbio | Cat: ml063154 |  |
| Mouse IL-6 | Proteintech | Cat: KE10007 |  |
| Mouse TNF-α | Proteintech | Cat: KE10002 |  |
| Mouse IL-10 | MULTI SCIENCES | Cat: EK210/4-96 |  |
| Human IL-35 | MULTI SCIENCES | Cat: EK135-96 |  |
| Reagents |  | | |
| Trizol | Life technologies | Cat: 15596018 |  |
| Linoleic acid (LA) | MCE | Cat: HY-N0729 |  |
| Conjugated linoleic acid | Sigma-Aldrih | 05507 |  |
| C9t11-Conjugated linoleic acid | MCE | Cat: HY-113162 |  |
| t10c12-CLA | Sigma-Aldrih | 2420-56-6 |  |
| Linolenic acid | MCE | Cat: HY-N0728 |  |
| Adenylsuccinic acid (ALA) | Sigma-Aldrih | 19046-78-7 |  |
| Eicosenoic acid (EA) | Sigma-Aldrih | 5561-99-9 |  |
| Conjugated linoleic acid ELISA Kit | KAMIYA BIOMEDICAL COMPANY |  |  |
| DC260126 (GPR40 inhibitor) | MCE | Cat: HY-101906 |  |
| AH-7614 (GPR120 inhibitor) | MCE | Cat: HY-19996 |  |
| YM-254890 (Gα4/11 inhibitor) | MCE | Cat: HY-111557 |  |
| Rimonabant (Gαi/o inhibitor) | Sigma | 168273-06-1 |  |
| Gallelin (Gβ and γ chain inhibitor) | Tocris Bioscience |  |  |
| Murine m-CSF | PEPROTECH | Cat: 315-02 |  |
| Human m-CSF | PEPROTECH | Cat: 300-25-10 |  |
| Monocolonal Anti-Ebi3(IL-35) blocking Antibody, clone V1.4C4.22 | Sigma | Cat: 3144423 |  |
| Recombinant IL-35 | PEPROTECH | 200-37 |  |
| Nuclear and Cytoplasmic Protein Extraction Kit | Beyotime | Cat: P0028 |  |
| Oligonucleotides for PCR |  |  |  |
| L.Reuteri-FW | BGI | 5’-ACCGAGAACACCGCGTTATTT -3’ |  |
| L.Reuteri-RW | BGI | 5’-CATAACTTAACCTAAACAATCAAAGATTGTCT -3’ |  |
| Oligonucleotides for CHIP-PCR |  | | |
| Mouse Ebi3-FW P1 | BGI | 5’-CCTTTCTCAGCTCTTACGTG-3’ |  |
| Mouse Ebi3-RW P1 | BGI | 5’-GCTGTGGGAAGCCATGGAACT-3’ |  |
| Mouse Ebi3-FW P2 | BGI | 5’-CTTGGGTGAGATTGTAAGTGG -3’ |  |
| Mouse Ebi3-RW P2 | BGI | 5’- CTGAGGCACAGGGAGAGGA-3’ |  |
| Human Ebi3-FW P1 | BGI | 5’-GGAGTCTCACTCTGTCACC -3’ |  |
| Human Ebi3-RW P1 | BGI | 5’- GTGGCAGGCACCTGTAATCC-3’ |  |
| Human Ebi3-FW P2 | BGI | 5’- CTCATCTGTGTCTCTCTCTG-3’ |  |
| Human Ebi3-RW P2 | BGI | 5’-AGGGACATGGAGGGAGAC -3’ |  |
| Oligonucleotides for siRNA |  | | |
| Mouse STAT1 siRNA sense(5’-3’) | Sangon Biotech | GGAGGUCUUUGUUCCCUUUTT |  |
| Mouse STAT1 siRNA antisense(5’-3’) | Sangon Biotech | AAAGGGAACAAAGACCUCCTT |  |
| Mouse STAT4 siRNA sense(5’-3’) | Sangon Biotech | CGUCCAUUGACAAGAAUGUTT |  |
| Mouse STAT4 siRNA antisense(5’-3’) | Sangon Biotech | ACAUUCUUGUCAAUGGACGTT |  |
| Human STAT1 siRNA sense(5’-3’) | Sangon Biotech | CCGCACCUUCAGUCUUUUCtt |  |
| Human STAT1 siRNA antisense(5’-3’) | Sangon Biotech | GAAAAGACUGAAGGUGCGtc |  |
| Human STAT4 siRNA sense(5’-3’) | Sangon Biotech | GCUGUUGCUAAAGGAUAAATT |  |
| Human STAT4 siRNA antisense(5’-3’) | Sangon Biotech | UUUAUCCUUUAGCAACAGCTT |  |
| Human GPR40 siRNA sense (5’-3’) | Sangon Biotech | CCUGGAGUGUGGUGCUUAAUC |  |
| Human GPR40 siRNA antisense (5’-3’) | Sangon Biotech | UUAAGCACCACACUCCAGGCA |  |
| Human GPR120 siRNA sense (5’-3’) | Sangon Biotech | CCUUCUUCUCCGACGUCAAGG |  |
| Human GPR120 siRNA antisense (5’-3’) | Sangon Biotech | UUGACGUCGGAGAAGAAGGGA |  |
| Bacteria strain |  |  |  |
| *Lactobacillus reuteri* | ATCC23272 | BioGaaia, Sweden |  |
| *Lactobacillus gasseri* | ATCC 33323 | BioGaaia, Sweden |  |
| *Lactobacillus animalis* | Bio-03681 | Biobw |  |
| *Lactobacillus hominis* | Bio-131681 | Biobw |  |

| Other reagents | | |
| --- | --- | --- |
| Ampicillin | Sigma | Cat: BP021 |
| Vancomycine | Sigma | Cat: V2002 |
| Neomycin sulfate | Sigma | Cat: N6386 |
| Metronidazole | Sigma | Cat: M3761 |
| Lipofectamin3000 | Invitrogen | Cat:L3000015 |
| ProteinA/G Magnetic Beads | MCE | Cat:HY-K02 |
| DMSO | Sigma | Cat:67-68-5 |
| MRS | 3M US | Cat: BP0275500 |
| QIAquick PCR Purification Kit | Qiagen | Cat:28104 |
| QuantiTect SYBR Green PCR Master Mix | Qiagen | Cat:208052 |
| Human Peripheral Blood Mononuclear Cells Separation Medium | Solarbio | Cat:P8680 |
| Human Peripheral Blood Lymphocyte Separation Medium | Solarbio | Cat:P8610 |
| FBS | Gibco | Cat:10099141 |
| Collagenase IV | Sigma | Cat: C5138 |
| DMEM | Gibco | Cat:11965118 |
| HBSS | Gibco | Cat:14170161 |
| Percoll | Solarbio | Cat: P8370 |
| Cell stimulation cocktail | ebioscience | Cat: 00-4975-03 |
| Foxp3 fix/perm buffer | Biolegend | Cat: 421403 |
| PMA | Sigma | Cat: 79346 |
| GolgiStop | BD Biosciences | Cat: 554724 |
| Permeabilization Buffer | eBioscience | Cat: 00-8333-56 |
| Lipopolysaccharides (LPS) | MCE | Cat: HY-D1056 |
